# Modular Design of Mesoporous Silica Nanoparticles Enables Bioimaging, Dual Chemotherapy, and Combinatorial Gene Silencing in Triple-Negative Breast Cancer

**DOI:** 10.1021/acsami.5c15253

**Published:** 2025-12-15

**Authors:** Laura P. Rebolledo, Punnya Anil Kumar Jeeja, Leyla Danai, Tamanna Binte Huq, Kirill A. Afonin, Juan L. Vivero-Escoto

**Affiliations:** † Department of Chemistry, 14727 University of North Carolina at Charlotte, Charlotte, North Carolina 28223, United States; ‡ Chemistry and Nanoscale Science Program, University of North Carolina at Charlotte, Charlotte, North Carolina 28223, United States; § Center for Biomedical Engineering and Science, University of North Carolina at Charlotte, Charlotte, North Carolina 28223, United States

**Keywords:** TNBC, MSN, NANPs, combinatorial RNAi, chemotherapeutics

## Abstract

Triple-negative breast cancer (TNBC) accounts for 10–15% of all breast cancers and remains the most aggressive subtype due to its resistance to standard chemotherapy. A key factor behind its resistance is the activation of antiapoptotic pathways, which help tumor cells evade drug-induced death. We present a modular platform for combinatorial therapy, simultaneously delivering different clinically approved chemotherapeutics and RNAi agents targeting multiple survival pathways in vitro. To ensure that therapeutic activation occurs only after cellular uptake, mesoporous silica nanoparticles were functionalized with cisplatin and gemcitabine via reduction-sensitive linkers, while surface modification with polyethylenimine enabled pH-responsive release of programmable RNA nanoparticles that, upon intracellular dicing, produce DS RNAs targeting the expression of Survivin and BCL-2. This optimized formulation achieved strong gene silencing, minimized immune activation, and enhanced cytotoxicity in TNBC cells compared to either treatment alone. The results highlight the potential of this combinatorial strategy to overcome chemoresistance by delivering multiple therapeutics that simultaneously target distinct survival pathways in cancer cells and can be readily modified and adapted to new targets.

## Graphical abstract

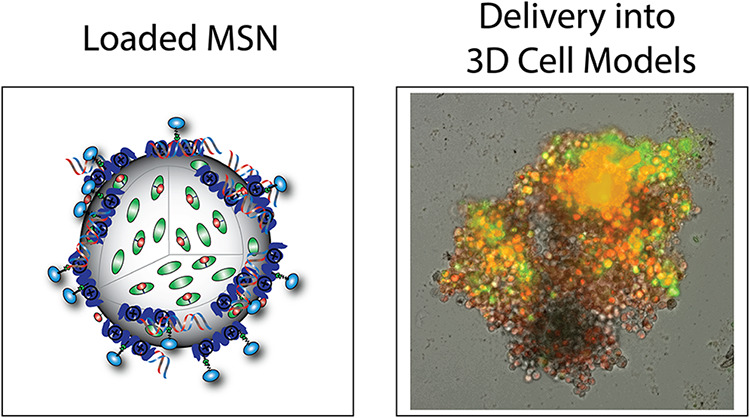

## Introduction

Drug resistance remains a major challenge for successful breast cancer treatment, with triple-negative breast cancer (TNBC) posing some of the most difficult clinical obstacles.^1–3^ Without targeted therapies, chemotherapy remains the primary option for advanced TNBC.^4^ Paclitaxel, doxorubicin, gemcitabine, and cisplatin are commonly used first-line chemotherapeutic agents that exert cytotoxic effects by inducing DNA damage, disrupting replication, and ultimately triggering cancer cell death.^5–8^ However, rapidly dividing TNBC cells often activate a range of survival pathways that enable them to evade or neutralize the effects of chemotherapy, diminishing treatment efficacy over time.^2,9^ Overcoming these limitations requires novel therapeutic approaches capable of concurrently targeting multiple intracellular pathways to circumvent or disrupt the molecular drivers of drug resistance.

Significant efforts have been dedicated to elucidating the mechanisms of TNBC chemoresistance and identifying actionable targets.^1^ One promising approach involves combining traditional chemotherapeutic agents with gene silencing therapies that specifically target resistance pathways, particularly antiapoptotic signaling controlled by proteins like Survivin and B-cell lymphoma 2 (BCL-2). TNBC tumors frequently express abnormally high levels of these proteins, contributing to unchecked proliferation and metastasis.^10–13^ Thus, targeting the expression of these biomolecules can enhance the therapeutic impact of chemotherapy while reducing the required dosage, thereby minimizing multidrug resistance, systemic toxicity, and concerning side effects.^14–17^ Interestingly, while some studies associate BCL-2 expression with improved survival in TNBC patients,^18^ others report that elevated survivin expression correlates with resistance to doxorubicin, gemcitabine, and cisplatin.^19–23^

RNA interference (RNAi) therapy has emerged as a powerful tool in this context, offering the ability to selectively silence genes involved in drug resistance and tumor progression, such as survivin and BCL-2.^24^ By disrupting these key resistance pathways, RNAi can improve the selectivity and potency of chemotherapy. However, to fully realize the potential of RNAi-based therapies, we must also develop effective delivery systems capable of transporting nucleic acid cargo into tumor cells, particularly in aggressive, treatment-resistant cancers like TNBC.

To achieve effective codelivery of multiple chemotherapeutics and dicer-substrate (DS) RNAs, nanoparticle-based systems have gained considerable interest.^25–27^ Among these, mesoporous silica nanoparticles (MSNs) offer key advantages over other platforms, including high surface area, tunable pore structure, and customizable surface chemistry.^28,29^ These features enable simultaneous loading of multiple therapeutic agents and controlled release in response to the tumor microenvironment.^30–34^ Furthermore, surface functionalization of MSNs with amine groups allows for electrostatic interactions with the phosphate backbone of DS RNAs, enabling stable complexation, delivery, and pH-assisted release of cargo.^35–39^

Our team has previously developed programmable nucleic acid nanoparticles (NANPs) that can incorporate various RNA therapeutics, including aptamers, antisense oligonucleotides, and DS RNAs. We designed NANPs in different shapes, sizes, and compositions to tune their biological behavior and delivery efficiency.^40–43^ We demonstrated that combining DS RNA-loaded fiber NANPs with doxorubicin-loaded MSNs enabled intracellular Dicer-assisted siRNA release and RISC-mediated gene silencing in cancer cell lines.^37^ These systems showed additive effects, promoting greater cell death than either therapy alone.

However, no study to date has investigated the integration of multiple chemotherapeutics, RNAi inducers, and imaging agents into a single therapeutic platform. In this work, we expand upon this concept by codelivering two DNA-targeting chemotherapeutic agents, gemcitabine and cisplatin, alongside fiber NANPs carrying multiple copies of DS RNAs. Upon intracellular dicing, these NANPs release DS RNAs targeting the antiapoptotic genes Survivin and BCL-2 (Figure 1).

**Figure 1:**
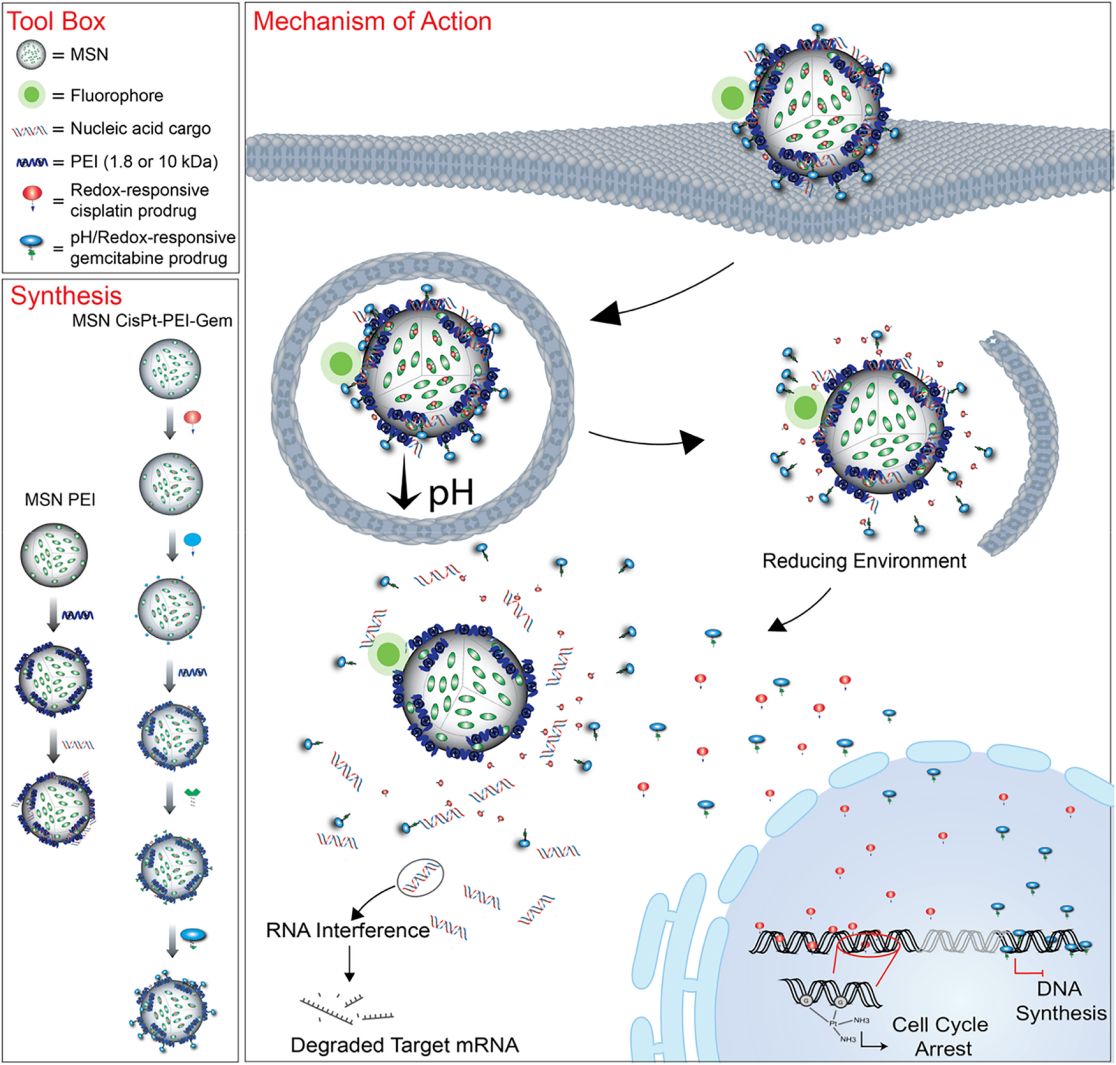
Schematic representation of MSN formulation steps, structural compositions of tested nanoparticles, and the proposed mechanism of action of complete formulations in triple-negative breast cancer (TNBC) cells.

To serve as an efficient scaffold and delivery vehicle for these functionalities, we developed a multifunctional MSN platform, functionalized with polyethylenimine (PEI) and phosphonate groups to enhance nucleic acid binding, protection, and intracellular delivery. Different PEI coatings were evaluated to identify the optimal formulation for delivery. The phosphonate modification was found to significantly enhance DS RNA binding and protect against nuclease-mediated degradation.

To enable controlled chemotherapeutic release, cisplatin (cisPt) and gemcitabine (Gem) were covalently conjugated to MSNs through reduction-sensitive linkers that respond to the elevated intracellular reductive conditions found in cancer cells. We then carried out extensive physicochemical characterization of the newly developed platform and optimized nucleic acid loading conditions to ensure complete cargo association and protection from nuclease degradation. As a model system, we demonstrated that the optimized nanoformulations effectively delivered DS RNAs designed for specifically targeting the green fluorescent protein (GFP), resulting in significant knockdown of GFP expression in MDA-MB-231/GFP reporter cells. Same experimental work was applied to further enable robust downregulation of survivin and BCL-2 protein levels when corresponding DS RNAs were introduced. To evaluate immunostimulatory potential and identify the least immunogenic design to be used for dual RNAi action, various NANP architectures were tested, with fiber NANPs shown to elicit the lowest immune response among them. Then, the fiber NANPs against BCL-2 and Survivin were designed, synthesized, and tested.

Cytotoxicity assays showed that the combination of MSN–cisPt–PEI–Gem with DS RNAs targeting Survivin and BCL-2 led to additive effects in MDA-MB-231 cells, significantly enhancing cell death beyond either treatment alone. This additive effect arises from complementary mechanisms: fiber NANPs carrying DS RNAs promote apoptosis via BCL-2 and Survivin inhibition, while cisPt and Gem independently exert cytotoxic effects. Importantly, the use of fiber NANPs preserved therapeutic efficacy while reducing immune activation. Overall, these results highlight the modularity and therapeutic potential of MSN-based nanocarriers for codelivering chemotherapeutics and gene-silencing RNAs in a targeted, immunotolerant, and effective manner, offering a promising strategy to overcome drug resistance and improve treatment outcomes in aggressive cancers such as TNBC.

## Results and Discussion

### Synthesis, Characterization, and Optimization of MSN-PEI and MSN-P-PEI

The loading efficiency and stability of therapeutic nucleic acids (e.g., DS RNAs) on MSNs depend on electrostatic interactions with positively charged PEI, which itself is electrostatically associated with the MSN surface.^33^ Two approaches can be used to synthesize MSN-PEI; the first one relies on the electrostatic interaction of PEI with silonates,^44^ and the second one requires functionalization of the MSNs with phosphonates (MSN-P).^33^ Herein, both approaches were tested and compared to identify the best alternative to carry DS RNA as described below. In addition, two different molecular weights of PEI were evaluated (MW = 1.8 kDa and 10 kDa).

MSNs were synthesized using a surfactant-template method.^33^ The physical properties of MSNs, including hydrodynamic diameter (D_h_), polydispersity index (PDI), Zeta-potential, surface area, and pore size, were determined (Figure 2A and SI Figure S1). The D_h_ of bare MSNs was found to be 107.6 ± 0.2 nm (PDI = 0.16 ± 0.03) with a zeta-potential of −37.2 ± 2.3 mV. The pore size and surface area were found to be 2.3 ± 0.1 nm and 799.77 m^2^/g, respectively. Further modification with PEI (1.8 kDa) or PEI (10 kDa) resulted in an increase in D_h_ and zeta-potential to be 117.9 ± 1.0 nm (PDI = 0.22 ± 0.04) and 34.0 ± 1.1 mV, or 112.0 ± 0.5 nm (PDI = 0.05 ± 0.01) and 34.0 ± 0.01 mV, respectively. The highly positive charge on PEI-coated MSN samples is due to the presence of free amines that are protonated in physiological conditions.^33,45^ The amount of amines on the surface of MSNs was determined using the ninhydrin test.^33^ This analysis showed that MSN-PEI (1.8 kDa) and MSN-PEI (10 kDa) contain 0.98 ± 0.07 and 0.95 ± 0.13 μmoles of free NH_2_ per mg of MSNs (Figure 2A). Once the MSNs were coated with phosphonate, the D_h_ and surface charge changed to 95.3 ± 0.75 nm (PDI = 0.14 ± 0.01) and −23.5 ± 1.9 mV, respectively. Modification with PEI (1.8 kDa) or PEI (10 kDa) resulted in an increase in D_h_ and zeta-potential to 116.3 ± 1.0 nm (PDI = 0.13 ± 0.04) and 34.6 ± 1.2 mV, or 131.5 ± 2.1 nm (PDI = 0.17 ± 0.12) and 31.0 ± 7.1 mV, respectively. Ninhydrin test analysis showed that MSN-P-PEI (1.8 kDa) and MSN-P-PEI (10 kDa) contain 1.06 ± 0.36 and 1.14 ± 0.40 μmoles of free amines per mg of MSNs (Figure 2A). TEM images of MSN-PEI and MSN-P-PEI (1.8 kDa) show no statistical difference in sizes and similar morphology (Figure 2B,C). The four samples MSN-PEI and MSN-P-PEI (1.8 or 10 KDa) present a good colloidal stability PDI < 0.2 with positively charged on the surface > +30 mV, and high content of free amines ∼1.0 μmoles of free NH_2_ per mg of MSNs. These are benchmark values for MSN materials to efficiently carry nucleic acids.

**Figure 2:**
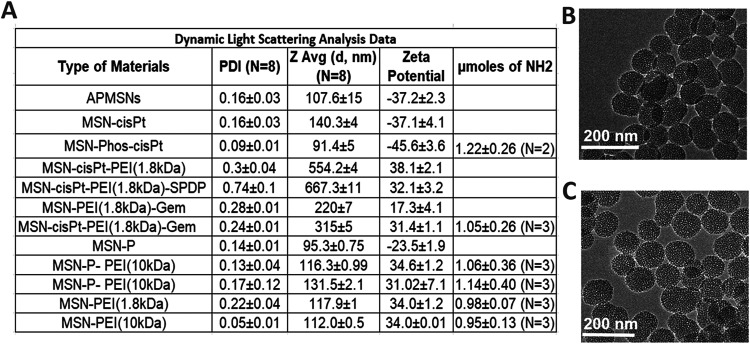
Characterization of different versions of MSN. (A) Table showing the hydrodynamic diameter, PDI, zeta-potential, and measured amounts of free amines (NH_2_) of different MSN versions. TEM images (Scale bar = 200 nm) of (B) MSN-PEI (1.8 kDa) 61.9 ± 4.1 nm (*n* = 60) and (C) MSN-P-PEI (1.8 kDa) 60.8 ± 4.6 nm (*n* = 60).

### Optimization of Nucleic Acid Binding to MSN-PEI and MSN-P-PEI

Our group has used MSN-P-PEI (1.8 kDa and 10 kDa) to enhance the drug loading capacity and introduce cationic sites for electrostatic interactions with nucleic acid cargo.^37,33,35^ In our prior work, we utilized MSN-P-PEI (10 kDa) to enable the efficient delivery of NANPs. However, the high molecular weight PEI is associated with cytotoxicity in vitro,^37,46,37,46^ necessitating the exploration of PEI with lower MW to develop biocompatible delivery systems. Therefore, we optimized the use of PEI-coated MSN based on two criteria: the molecular weight of PEI and the presence of phosphonate on the surface. First, binding assays were performed by complexing MSN-PEI or MSN-P-PEI (10 or 1.8 kDa) with a fluorescently labeled 27-bp DNA duplex at varying N/P ratios (where N corresponds to the number of available amine groups on the MSN surface and P represents the number of phosphate groups on the nucleic acid backbone). A fluorescently labeled DNA duplex was used as a cost-effective model system, designed to mimic the structural features of Dicer-substrate (DS) RNA.

Binding was assessed using electrophoretic mobility shift assays (EMSAs). Effective binding was indicated by a reduction or complete disappearance of the free DNA band during agarose gel electrophoresis, as the interaction between the negatively charged nucleic acid and the PEI-coated MSNs led to retention of the complex in the loading well (Figure 3A). As shown in Figure 3B, MSN-P-PEI (1.8 or 10 kDa) demonstrated the strongest binding to nucleic acids at an N/P ratio of 4, then MSN-PEI (1.8 kDa) or MSN-PEI (10 kDa) at an N/P ratio of 5 and 8, respectively. Notably, for MSN-PEI (1.8 kDa) formulations, faint streaking was observed in the gel after the free duplex disappeared, and complexes were retained in the wells. This may indicate lower binding stability, suggesting that phosphonate groups facilitate stronger and more uniform interactions between MSNs and nucleic acids. Importantly, no substantial difference was observed between the binding efficiency of MSN-P-PEI (1.8 kDa) and MSN-P-PEI (10 kDa) when complexed with Alexa488-labeled DNA duplexes. We confirmed that RNA duplexes of the same length demonstrate comparable binding efficiency to their DNA counterparts (Figure S2). Because both MSN-P-PEI (1.8 kDa) and MSN-P-PEI (10 kDa) formulations performed similarly, we chose to carry them forward for the next set of optimization studies with nucleic acids. For clarity, the phosphonate (P) modification will not be explicitly labeled in subsequent figures, but all MSN-PEI formulations used hereafter include phosphonate functionalization.

**Figure 3:**
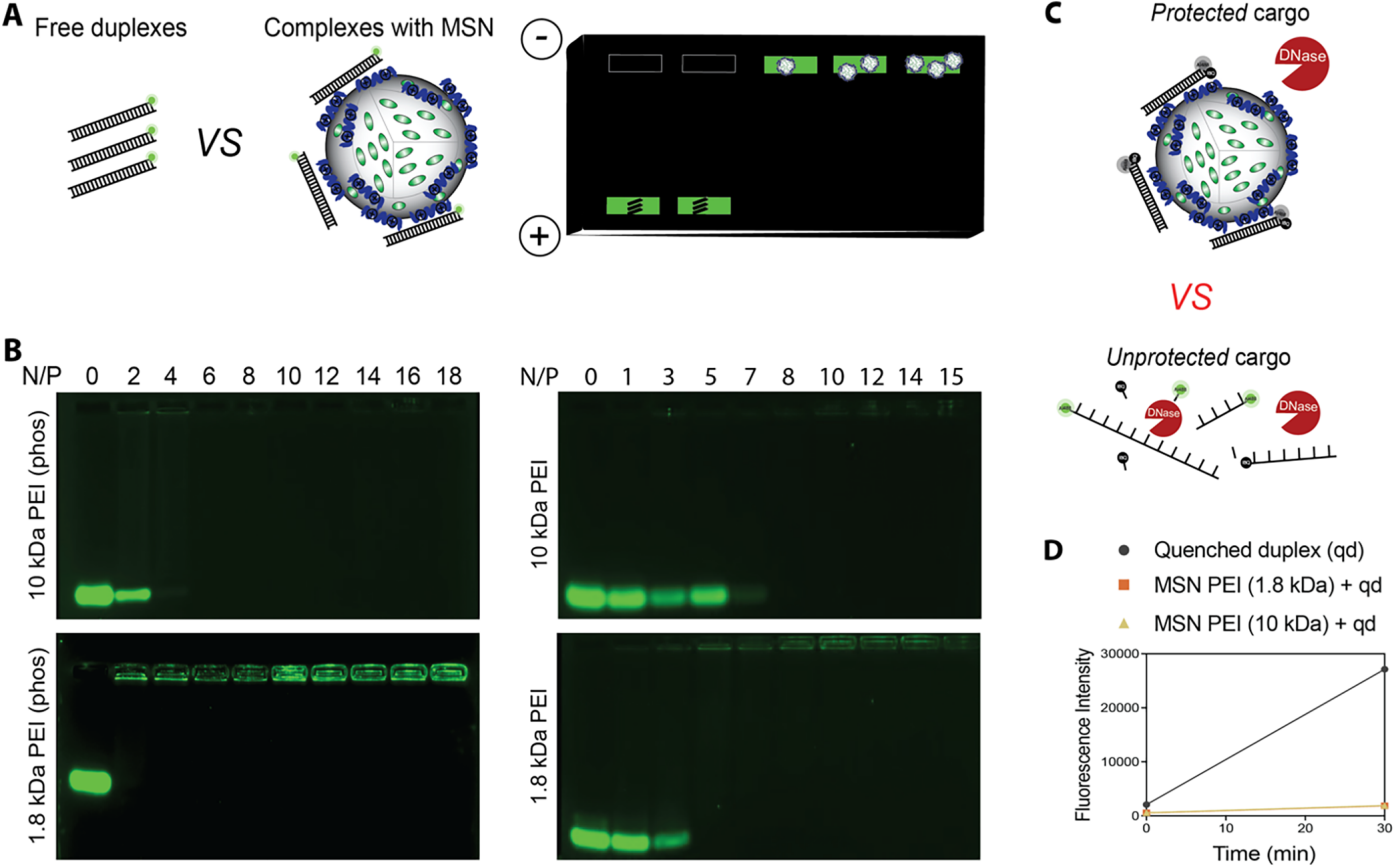
Evaluation of nucleic acid binding and nuclease protection by PEI-functionalized MSNs. (A) Schematic of the binding assay in which MSNs are complexed with a 27-bp Alexa Fluor 488-labeled DNA duplex. Binding is indicated by the disappearance of the free DNA band and retention in the gel well. (B) Agarose gel showing binding efficiency of phosphonate-modified (P) vs nonphos MSNs functionalized with 1.8 kDa or 10 kDa PEI at varying N/P ratios. MSN-P show stronger binding at lower N/P ratios. (C) Schematic of the nuclease protection assay. Fluorescence is activated upon DNA degradation; protected duplexes remain quenched. (D) DNase protection results for MSN-P-PEI formulations. Both MSN-PEI-1.8 kDa and 10 kDa protect DNA from enzymatic degradation. *All MSN-PEI formulations used include phosphonate functionalization, though not explicitly labeled*.

### Nuclease Protection Assay

To confirm that the MSN-PEI carriers protect bound nucleic acid cargo from nuclease degradation, MSN-PEI (1.8 or 10 kDa) formulations were complexed with a quenched, fluorophore-labeled DNA duplex and exposed to DNase. In this model system, fluorescence is unquenched upon DNA digestion, providing a readout of nuclease activity (Figure 3C).^47–50^ The DNA duplex complexed with MSN-PEI (1.8 or 10 kDa) remained largely intact following DNase treatment, as evidenced by minimal fluorescence activation (Figure 3D). We had previously shown that PEI 10 kDa efficiently protects DNA from enzymatic degradation.^37^ These results are consistent with previous reports and indicate that the MSN-PEI formulation effectively shields nucleic acids from enzymatic degradation, supporting its potential for stable extracellular transport and successful intracellular delivery.

### Specific Gene Silencing Studies

MSNs have been demonstrated to enter cells via endocytosis and subsequently undergo trafficking along the endolysosomal route.^37^ The efficacy of gene silencing relies on the efficient release of DS RNA in the cytoplasm. Therefore, we evaluated the colocalization of AF488-labeled DNA duplex delivered by MSN-PEI carriers inside the cells and lysosomes by staining the cells with DAPI and LysoTracker Red. Confocal microscopy images revealed that AF488-labeled DNA duplex-MSN-PEI is colocalized within lysosomes (Figure 4A). However, some instances showed nanoparticles dispersed outside of any identifiable organelles, suggesting lysosomal escape—likely driven by the “proton sponge effect” attributed to PEI polymers. These findings collectively support the delivery of AF488-labeled DNA duplexes into the cytoplasmic compartment by MSN-PEI carriers.

**Figure 4:**
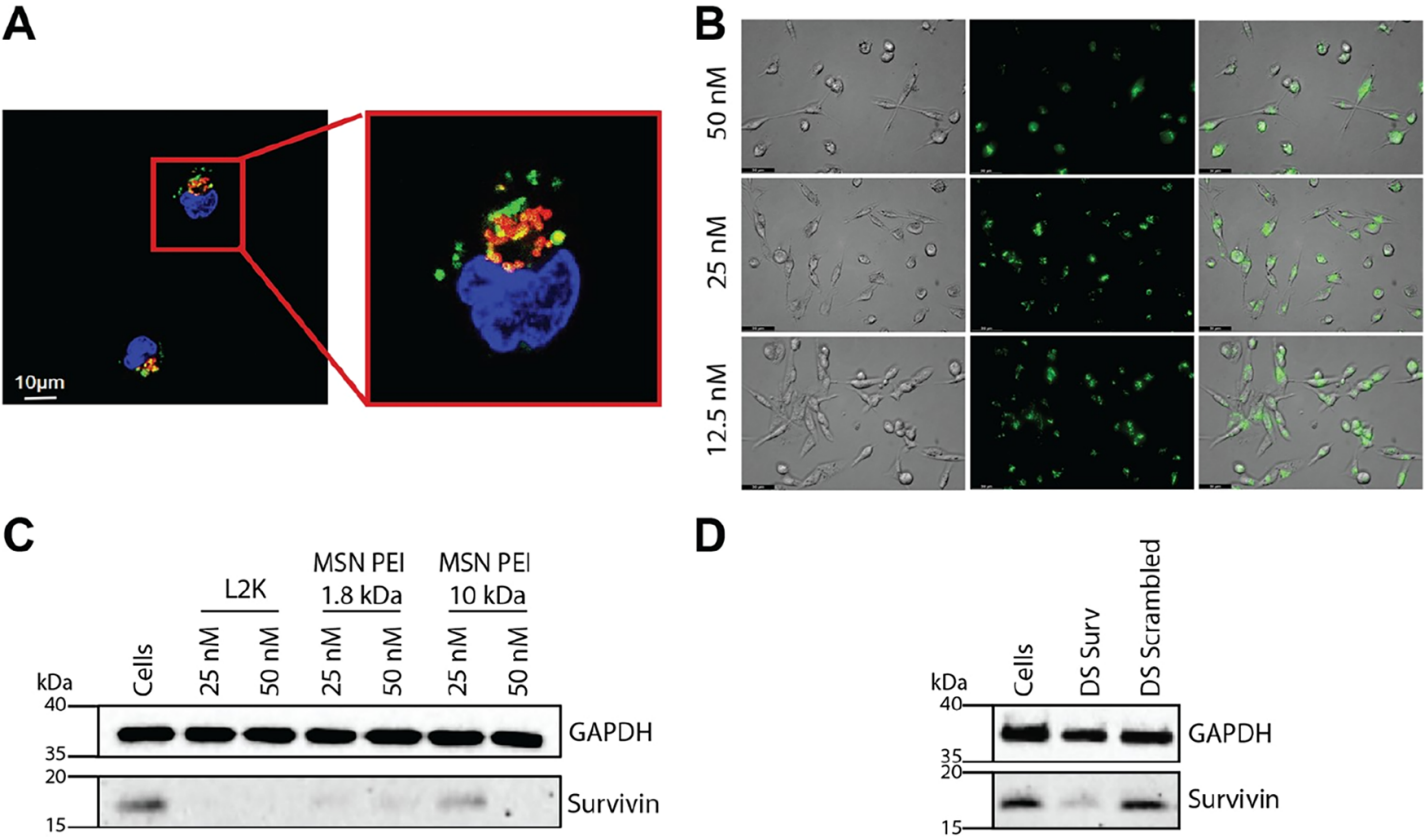
Cellular uptake, gene silencing efficiency, and protein downregulation were evaluated using functionalized MSNs. (A) Confocal images show cellular uptake of AF488-labeled DNA duplex delivered by TRITC-labeled MSN-PEI carriers in MDA-MB-231 cells. Cells were treated with MSN-PEI (1.8 kDa) complexes at an N/P ratio of 10 using 10 nM AF488-labeled DNA duplex. (B) Representative fluorescent images show cellular uptake of AF488-labeled DNA duplex delivered by MSN-PEI (1.8 kDa) at N/P ratio 10 and using DNA duplex concentrations of 12.5, 25, and 50 nM in MDA-MB-231 cells (Scale bar = 50 μm; 40× magnification). (C) Western blot analysis demonstrating survivin protein downregulation in MDA-MB-231 cells following treatment with Survivin targeting dicer substrate RNA complexed with MSN-PEI (1.8 and 10 kDa) at 25 nM and 50 nM. (D) Western blot analysis showing Survivin protein expression in MDA-MB-231 cells treated with DS Surv or Scrambled DS RNA complexed with MSN-PEI at an N/P ratio of 10 and a total nucleic acid concentration of 50 nM.

To assess the cellular uptake of nucleic acid components, AF488-labeled double-stranded (ds) DNA bound to MSN-P-PEI (1.8 kDa) at an N/P ratio of 10 was used to visualize internalization in MDA-MB-231 cells (Figure 4B). Furthermore, to evaluate whether the MSN-PEI (1.8 or 10 kDa) formulations can effectively deliver RNAi inducers inside cells, DS RNAs targeting GFP were complexed with the nanoparticles and transfected into MDA-MB-231 cells engineered to express GFP. The complexes were prepared at varying N/P ratios, selected based on earlier binding assays to ensure optimal loading and interaction. Following transfection, GFP silencing efficiency was quantified by flow cytometry to assess the functional delivery of DS RNAs. All tested N/P ratios demonstrated efficient gene silencing (SI Figure S9). For the MSN-PEI (1.8 kDa), a higher degree of GFP gene silencing was observed when the N/P ratio increased, and when the RNA concentration was raised from 25 to 50 nM. Although these increases were not statistically significant, the consistent trend suggests improved delivery and silencing potential at higher formulation parameters. In contrast, the MSN-PEI (10 kDa) formulation did not demonstrate a significant change in silencing efficiency across varying N/P ratios or RNA concentrations. The lack of a notable difference indicates that, under the tested conditions, increasing the formulation parameters does not enhance silencing efficacy for this higher molecular weight PEI. These results suggest that the lower-molecular-weight PEI can support effective RNA delivery while offering a potentially improved safety profile.

Western blot analysis (Figure 4C) confirmed that the functionalized MSN-PEI carriers effectively deliver Survivin-targeting DS RNA, resulting in a measurable reduction of Survivin protein levels in MDA-MB-231 cells. Both MSN-PEI (1.8 kDa) and MSN-PEI (10 kDa) formulations induced knockdown at DS RNA concentrations of 25 and 50 nM. Notably, MSN-PEI (1.8 kDa) achieved more efficient Survivin downregulation at both concentrations compared to MSN-PEI (10 kDa), suggesting enhanced delivery performance even at lower doses. We further demonstrated the specificity of DS Surv by using a DS Scrambled, which did not show any reduction of Survivin expression (Figure 4D). Although both Survivin and BCL-2 DS RNAs were evaluated in cytotoxicity assays, Survivin was selected for protein-level validation due to its well-characterized role in TNBC chemoresistance. BCL-2 knockdown has been previously demonstrated by our group,^37^ and was therefore not repeated here at the protein level. Full Western blots are shown in SI Figure S8. Based on these findings, MSN-PEI (1.8 kDa) at an N/P of 10, was selected for continued use on the rest of the work, as it maintained efficacy comparable to MSN-PEI (10 kDa) while reducing potential cytotoxicity.

### Synthesis and Characterization of MSN-cisPt-PEI-Gem

The synthesis of MSN-cisPt-PEI-Gem was carried out through a multistep approach outlined in Figure 1.^33^ First, the prodrugs of cisPt and Gem were synthesized using a two-step process adapted from reported methodologies with slight modifications from our lab to load into the MSNs.^33^ Use of prodrugs instead of free cisPt or Gem would reduce off-target toxicities and are more consistent and reproducible in loading capacity.^51,52^ It also ensures stimulus-responsive release of drugs in the target-specific tumor site due to the low pH and reducing environment compared to healthy tissue.^33^ cisPt was modified through a two-step process to form cisPt prodrug (SI Figure S1). The prodrug was further loaded to MSN via a coupling reaction mediated by 1-ethyl-3-(3-(dimethylamino)­propyl) carbodiimide hydrochloride (EDC). The percentage of cisPt loaded into the MSN was determined using atomic absorption spectroscopy (AAS) and found to be 21.1 ± 1.2% wt (*n* = 4). The physical characterization of MSN-cisPt showed a D_h_ of 140.3 ± 4.0 nm (PDI = 0.16 ± 0.03) and a zeta-potential of −37.1 ± 4.1 mV (Figure 2A). This material was modified with phosphonates to render it stronger negatively charged on the surface. The D_h_ of MSN-cisPt-P was 91.4 ± 5.0 nm (PDI = 0.09 ± 0.01) with a zeta-potential of −45.6 ± 3.6 mV. MSN-cisPt-P was further coated with PEI (1.8 kDa) to afford MSN-cisPt-PEI. The D_h_ of the material increased to 554.2 ± 4.0 nm (PDI = 0.3 ± 0.04) with a zeta-potential of 38.1 ± 2.1 mV. The change in the surface charge is expected due to the PEI coating. The increase in Dh, can be associated with partial aggregation due to the modification of MSNs with cisPt and PEI. Nonetheless, the PDI values remain within an acceptable range, confirming that the formulations are colloidally stable. Quantification of free amine groups by the ninhydrin test was found to be 1.22 ± 0.18 μmoles (*n* = 2) of amine groups per mg of MSNs. To afford the multichemotherapy material, MSN-cisPt-PEI-Gem, a Gem prodrug was synthesized in a two-step process (SI Figure S3). The MSN-cisPt-PEI was further modified with SPDP (*N*-succinimidyl 3-(2-pyridyldithio)­propionate) linker to load Gem prodrug via disulfide exchange (MSN-cisPt-PEI-Gem). The percentage loading of Gem was calculated to be 18.7 ± 0.7% wt (*n* = 8) according to UV–vis spectroscopy. The D_h_ and zeta-potential of the final material were found to be 315 ± 5 nm (PDI = 0.24 ± 0.014) and 31.4 ± 1.1 mV. Partial aggregation of MSN-cisPt-PEI-Gem is most likely due to the presence of Gem on the surface of the nanoparticles. However, the zeta-potential still indicates free amines available for further interaction with nucleic acids. Ninhydrin analysis shows that there are 1.05 ± 0.26 μmoles of amine groups per mg of MSNs in MSN-cisPt-PEI-Gem. The reduction in free amines following functionalization with Gem prodrug accounts for a loading variation ranging from 9.2 to 20.7 wt %; however, the value determined by UV–vis analysis is considered more accurate.

### Cytotoxic Evaluation of MSN Formulations and the Combination with DS RNAs Targeting Survivin and BCL-2

The cytotoxic effect of three different MSN materials was evaluated using the MTS assay in MDA-MB-231 cell line. The drug-response plot was determined for MSN-cisPt-PEI, MSN-PEI-Gem and MSN-cisPt-PEI-Gem (SI Figure S3). Based on these plots the EC_50_ was calculated to be 538.7 μg/mL (304.2 μM of cisPt), 38.5 μg/mL (23.1 μM of Gem) and 21.1 μg/mL (12.6 μM of Gem and 11.9 μM of cisPt) for MSN-cisPt-PEI, MSN-PEI-Gem and MSN-cisPt-PEI-Gem, respectively. The cytotoxic results demonstrated that by combining both Gem and cisPt in the same nanoparticle, an additive effect is obtained. The amounts of cisPt and Gem required to achieve the EC_50_ were reduced by 25-fold and 2-fold, respectively. It is important to point out that MSN-PEI does not exhibit high cytotoxicity at concentrations even as high as 500 μg/mL.

For the combinatorial studies with MSN formulations and DS RNAs targeting Survivin and BCL-2, concentrations below the EC_50_ of MSN-cisPt-PEI-Gem were employed to clearly evaluate the contributions of DS RNA and the combined therapeutic effects. To test the therapeutic potential of the MSN-cisPt-PEI-Gem material in combination with nucleic acids, Survivin- and BCL2-targeting DS RNAs were complexed at an N/P ratio of 10 and tested in MDA-MB-231 cells. Cytotoxicity was assessed at various DS RNA concentrations (10, 15, 25, 50, and 100 nM). Higher concentrations of MSN-cisPt-PEI-Gem resulted in masking the silencing effect of DS RNA (SI Figure S4). Nevertheless, the concentrations of 10 and 15 nM were the most optimal for the combination. Only Survivin DS RNA with the carrier MSN-PEI reduced cell viability to 63.3 ± 4.6 and 66.19 ±  5.2% for 10 and 15 nM, respectively (Figure 5). This confirms that the Survivin-loaded MSNs are functionally active and capable of slowing down cell growth even in the absence of chemotherapeutics.^53^ MSN-cisPt-PEI-Gem at 10 and 15 nM DS RNA showed enhanced cytotoxicity with Survivin, reducing viability to 52.4 ±  5.2 and 45.7 ±  9.2% compared to 74.5 ±  1.2 and 66.2 ± 8.8% for the non-DS RNA-loaded version. The results at 10 and 15 nM for the MSN-CisPt-PEI-Gem with and without Survivin showed statistical difference. This shows that the combined effects of DS RNA-chemotherapeutic formulations performed better than each individual approach. Similar results were observed in BCL-2 DS RNA as well. MSN-PEI-BCL2 reduced cell viability to 65.3 ±  9.7 and 68.2 ± 9.3% for 10 and 15 nM DS RNA concentration, respectively. In addition, a reduced cell viability of 49.2 ±  5.2 and 48.5 ± 4.3% was observed with BCL2 DS RNA-loaded MSN-cisPt-PEI-Gem at 10 and 15 nM. This cell viability reduction is significant compared with the non-DS RNA-loaded version. The DS RNA-loaded MSN-PEI formulation shows that the DS RNA (Survivin or BCL-2) successfully targets the gene-specific silencing, leading to cell death or cell growth inhibition. The addition of chemotherapeutics further reduces the cell viability, showing the combinatorial effect of DS RNA-drug formulations loaded to the MSN carrier. This additional 25–30% reduction highlights the cooperative effect of combining DS RNA-mediated gene silencing with dual chemotherapeutic delivery. The mRNA expression of BCL-2 using the MSN-PEI platform loaded with doxorubicin as the chemotherapeutic agent has been previously reported in our earlier publication.^37^ Furthermore, the expression of Survivin mRNA in TNBC and other breast cancer models has also been well-documented in previous studies.^54–58^

**Figure 5:**
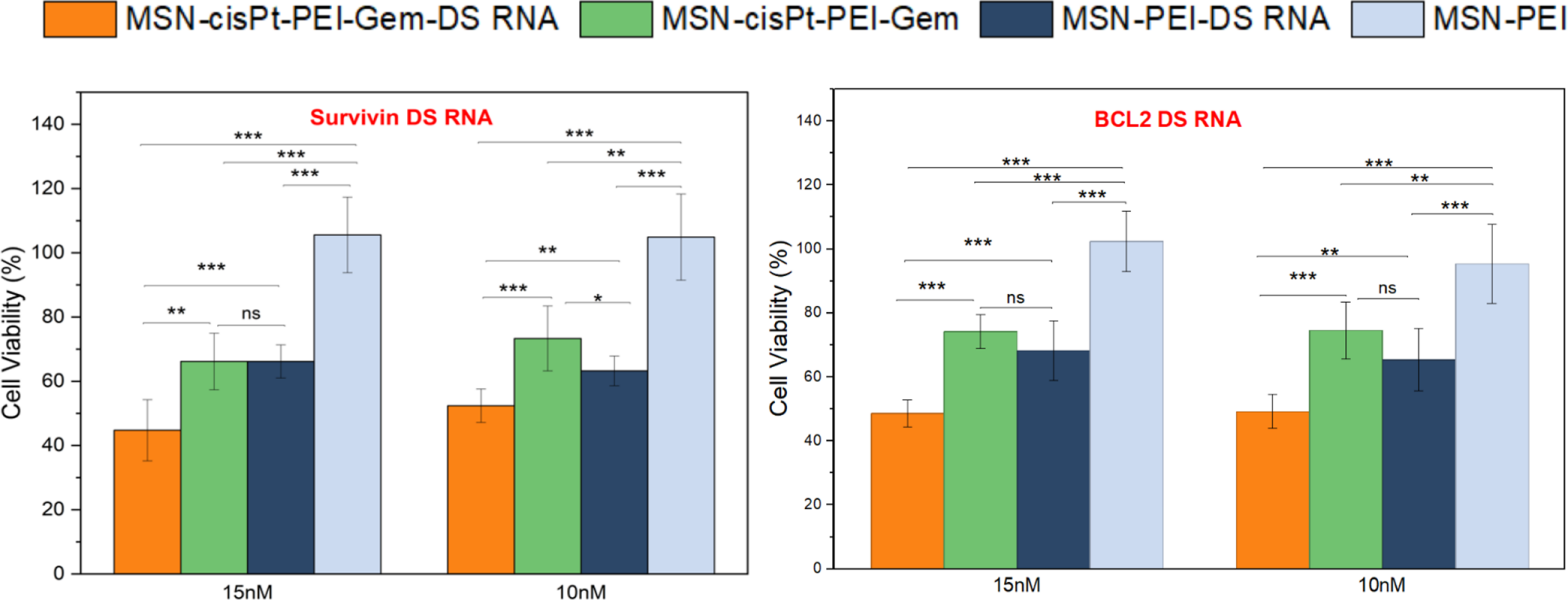
Cell viability analysis of MSN-PEI-DS RNA formulations in MDA-MB-231 cells. Error bars represent mean ± SD from three biological replicates (*n* = 3), *­(*p* < 0.05), **­(*p* < 0.01), ***­(*p* < 0.001), ****­(*p* < 0.0001).

### Immunostimulation and Cytotoxicity by NANPs Complexed with MSNs In Vitro

To assess the immunostimulatory potential of MSNs complexed with different RNA NANPs, THP1-Dual and HEK-Lucia RIG-I reporter cells were used to evaluate the activation of the interferon regulatory factor (IRF). Two representative architectures, RNA cubes (3D) and RNA fibers (1D), were compared. As shown in Figure 6, in THP1-Dual cells, an increase in IRF activation was observed when RNA cubes were complexed with MSN-PEI (10 kDa). A modest, though not statistically significant, increase in activation was also noted with RNA cubes complexed to MSN-PEI (1.8 kDa). In contrast, no measurable IRF activation was detected for complexes containing RNA fibers or DS RNA with either MSN-PEI formulation. A similar trend was observed in HEK-Lucia RIG-I cells. RNA cubes complexed with MSN-PEI (10 kDa) induced significant IRF activation compared to those complexed with MSN-PEI (1.8 kDa), while complexes with RNA fibers or DS RNAs failed to elicit any detectable immune response. These findings are consistent with previous observations that fiber NANPs induce minimal immune activation, a key characteristic that supports their potential use in applications where reduced inflammation and improved safety profiles are critical.^39,59–61^

**Figure 6:**
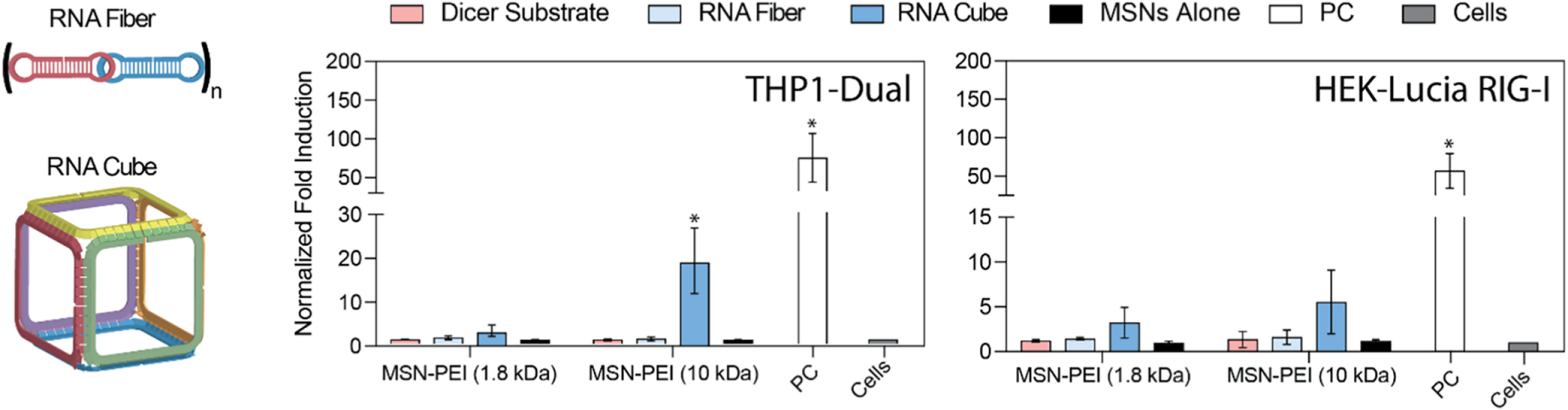
Immunostimulation studies using THP1-Dual and HEK-Lucia RIG-I reporter cells treated with MSN-PEI (1.8 kDa or 10 kDa) complexed with different RNA nanostructures (dicer substrate RNA, RNA fiber, or RNA cube) and their respective controls. (*n* = 3; mean ± SD; * (*p* < 0.05)).

### Cytotoxic Evaluation of the Combination of MSN-cisPt-PEI-Gem and Fiber NANPs Targeting BCL-2 and Survivin

The successful assembly of all DS RNAs and corresponding fiber NANPs (fNAs) targeting Survivin, BCL2, and their combination was confirmed, as shown in Figure S5. Each construct exhibited the expected structural formation, validating the design and synthesis protocols used for generating functional nucleic acid nanoparticles.

The cytotoxicity of the MSN formulations with BCL-2 and Survivin functionalized fNAs were tested in MDA-MB-231 cells at an N/P ratio of 10. Both the BCL2-fNAs and Surv-fNAs were evaluated at 10 and 1 nM. Concentration below 10 nM was tested to see if the combination behaves better at a lower concentration of the fNAs compared to the DS RNA due to higher number of phosphates per mole (Figure 7). Surv-fNAs with only MSN-PEI showed reduced toxicity of 69.9 ± 7.3 and 77.1 ± 9.8% at 10 and 1 nM, respectively. The reduction in cell viability due to Surv-fNAs is similar for DS RNA at 10 nM; nevertheless, at 1 nM Surv-fNAs showed a lower reduction (SI Figure S6). Surv-fNAs with MSN-cisPt-PEI-Gem showed a viability of 59.9 ± 4.4 and 70.4 ± 8.1% at 10 and 1 nM. The cell viability at 10 nM is lower than the one obtained with MSN-cisPt-PEI-Gem, but not statistically different. In addition, the cell viability of Surv-fNAs is not statistically different from the corresponding DS RNA at 10 nM (SI Figure S6). However, the reduction in cell viability at 1 nM of Surv-fNAs is better than DS RNA-loaded MSN-cisPt-PEI-Gem at 10 nM. This shows that the Surv-fNA combined with chemotherapeutics at a concentration of 1 nM performs better than the chemotherapeutics alone and is similar to 10 nM fNA and DS RNA. In the case of BCL2-fNAs with MSN-PEI showed reduced toxicity of 63.4 ± 12.8 and 72.5 ± 13.2% at 10 and 1 nM, respectively. The reduction in cell viability due to BCL2-fNAs is similar for DS RNA at 10 nM; nevertheless, at 1 nM BCL2-fNAs showed a lower reduction (SI Figure S6). BCL2-fNAs with MSN-cisPt-PEI-Gem showed a viability of 53.3 ± 6.5 and 64.9 ± 7.3% at 10 and 1 nM, which demonstrated a reduction compared with MSN-cisPt-PEI-Gem, 60.1 ± 10.9 and 83.2 ± 8.9% at same concentrations. Nevertheless, BCL2-fNAs at only 1 nM performed better than MSN-cisPt-PEI-Gem alone. Similar to Surv-fNAs, our data showed that the BCL2-fNAs combined with chemotherapeutics at a concentration of 1 nM outperformed the chemotherapeutics alone and performed similarly to 10 nM BCL2-fNAs and BCL2-DS RNAs.

**Figure 7:**
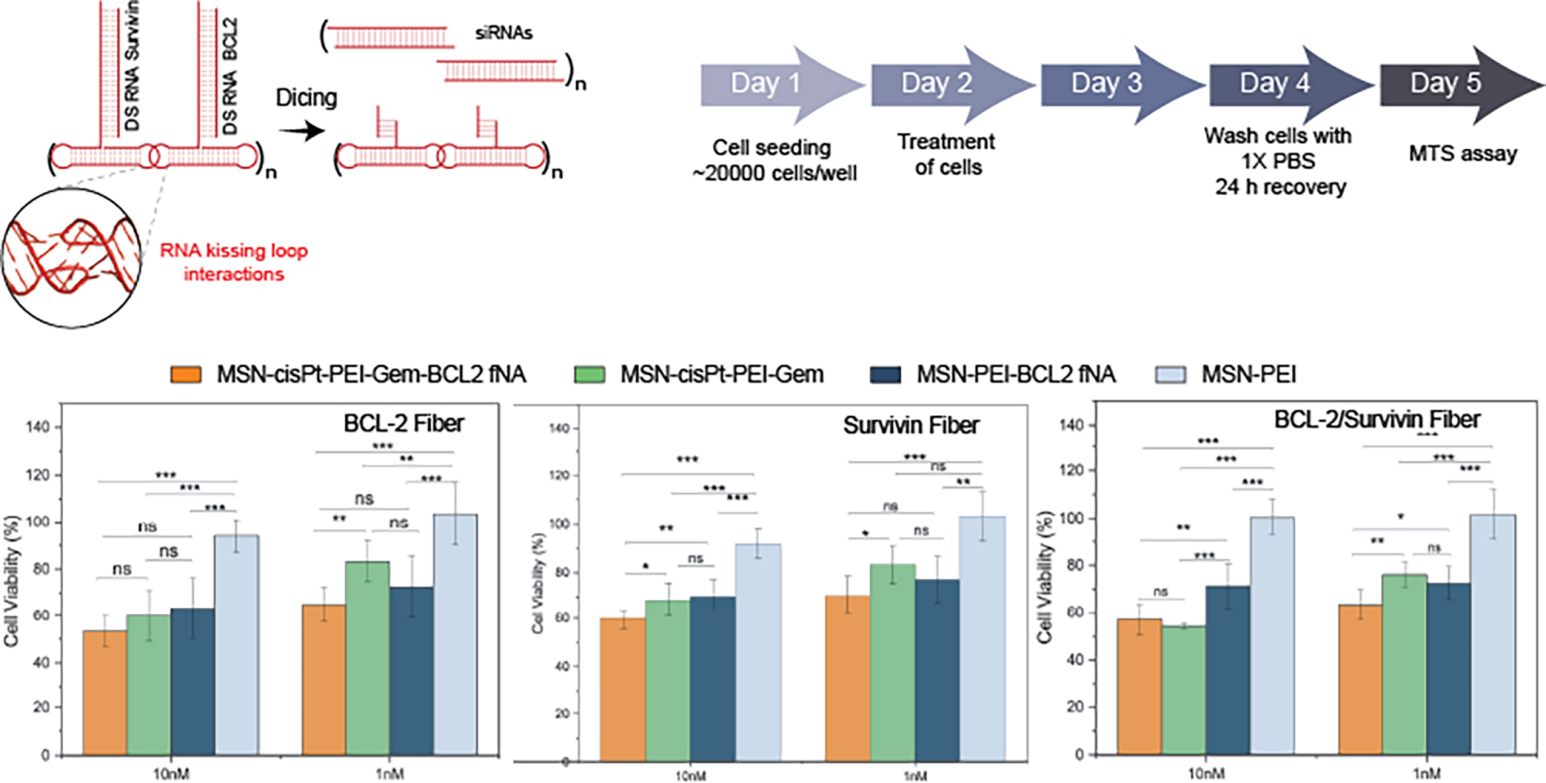
Schematic representation of functional RNA fibers, protocols, and results. Cell viability analyses of fibers at 1 and 10 nM. Error bars represent mean ± SD from three biological replicates (*n* = 3), *­(*p* < 0.05), **­(*p* < 0.01), ***­(*p* < 0.001), ****­(*p* < 0.0001).

The Surv-BCL2-fNAs were also tested at 10 and 1 nM to evaluate the therapeutic effects on cell viability after treatment. The Surv-BCL2-fNAs with the MSN-PEI showed a reduced viability of 71.2 ± 9.3 and 72.52 ± 9.31% at 10 and 1 nM, respectively. The reduction of cell viability was similar to that observed for single Surv-fNAs or BCL2-fNAs (SI Figure S7). In the case of combination with chemotherapeutics, a cell viability of 57.0 ± 6.6 and 63.2 ± 6.4% was observed at 10 and 1 nM, respectively. These results are statistically similar to those obtained for single Surv-fNAs or BCL2-fNAs. This shows that the combination of Surv and BCL-2 DS RNAs in the same fNAs does not provide any therapeutic advantage.

### New Approach Methodologies

The use of New Approach Methodologies (NAMs), such as 3D cell culture models and organ-on-a-chip platforms, provides advanced and physiologically relevant systems to evaluate the efficacy and safety of novel therapeutic strategies while reducing reliance on animal testing.^62^ These models better recapitulate the tumor microenvironment, including gradients of oxygen, nutrients, and drug penetration, offering more predictive insights into treatment responses compared to traditional 2D monolayer cultures.^63–65^

In this study, MDA-MB-231 3D spheroid models were used to evaluate the therapeutic potential of chemotherapeutic drug-loaded MSNs complexed with each respective NANPs. As shown in Figure 8A, these results demonstrate efficient cellular internalization of TRITC-MSN-PEI and confirm successful codelivery of Al488-labeled DS DNA within the 3D spheroid model. Furthermore, Figure 8B illustrates the comparative response of spheroids under different treatment conditions. The untreated control spheroids at 0 and 24 h, where spheroid growth and compaction are evident over time. In contrast, spheroids treated with MSN–cisPt–PEI–Gem–RNA exhibit a noticeable reduction in spheroid size, indicating enhanced cytotoxicity and impaired spheroid integrity. Specifically, the Surv/BCL-2 fiber, which targets key antiapoptotic pathways, contributed to a significant decrease in spheroid viability when combined with chemotherapeutic agents. The observed reduction in spheroid size supports the combinatorial effect of the formulation, which acts through multiple mechanisms, enhancing therapeutic efficacy while potentially overcoming resistance mechanisms often seen in triple-negative breast cancer cells. The combinatorial formulation integrates two chemotherapeutics (cisPt and Gem), the MSN-based delivery system, and nucleic acid nanoparticles targeting BCL-2 and Survivin. In this design, these complementary mechanisms act in a combinatorial manner to enhance cytotoxic efficacy, disrupt spheroid integrity. This contributes to the potent antitumor effect observed in the spheroid model, demonstrating the value of integrating NAMs such as 3D cultures to more accurately assess nanotherapeutic efficacy prior to in vivo validation.

**Figure 8:**
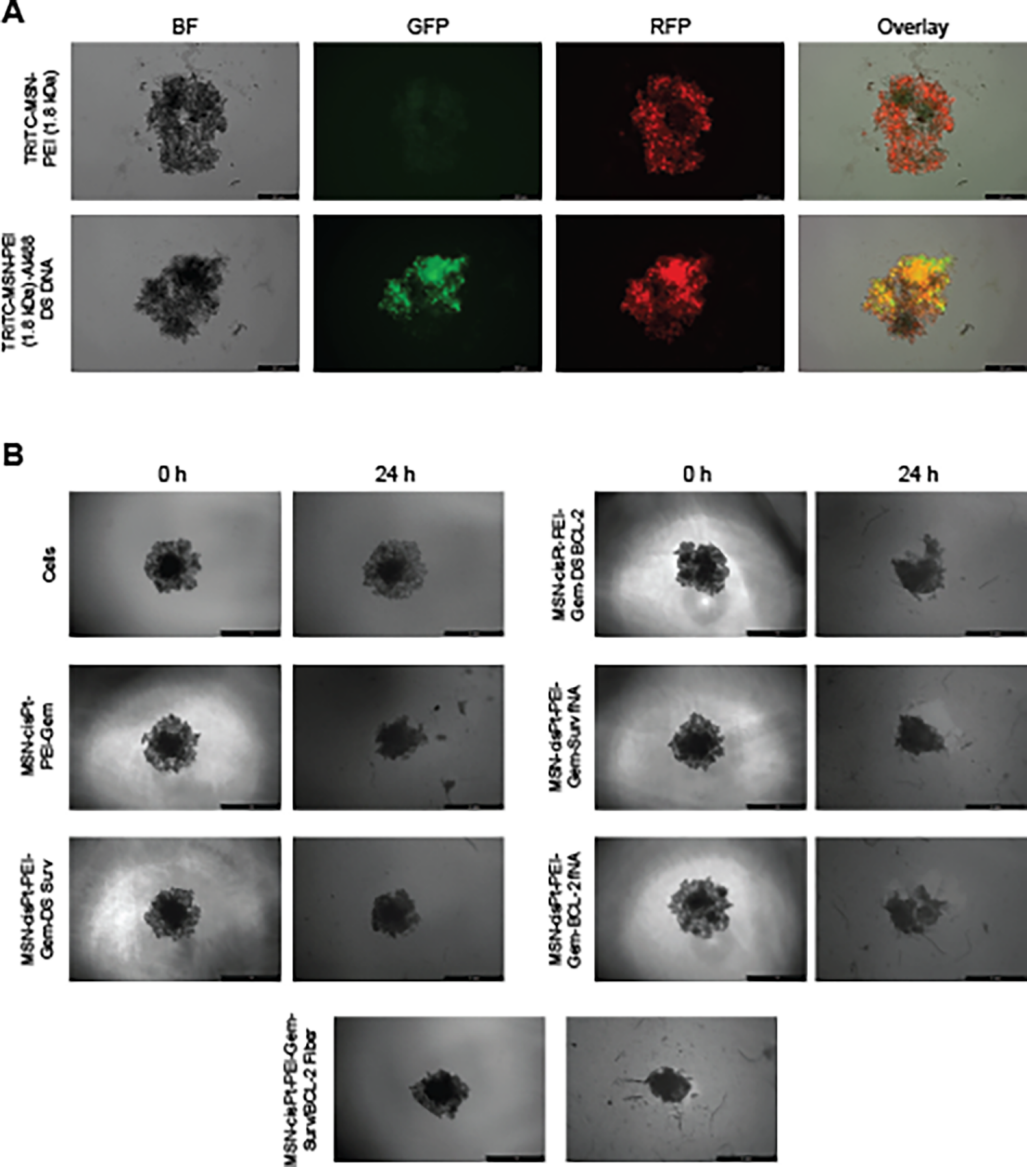
(A) Representative images of 3D MDA-MB-231 spheroid models showing cellular uptake of TRITC-labeled MSN-PEI (1.8 kDa) and TRITC-MSN-PEI (1.8 kDa) complexed with Al488-DS DNA. Images were captured 24 h post-treatment, illustrating intracellular localization of nanoparticles and Al488-DS DNA complexes. Scale bar = 250 μm. (B) Representative images of 3D MDA-MB-231 spheroid models showing treatment response over time. Untreated spheroids (0 h) served as the control to assess baseline morphology and size. Images at 24 h post-treatment illustrate the effects of chemotherapeutic drug-loaded MSNs complexed with different NANPs on spheroid integrity and size reduction. Scale bar = 1 mm.

## Conclusion

This study presents a comprehensive approach to designing, optimizing, and evaluating MSNs functionalized with PEI for the codelivery of nucleic acids and chemotherapeutic agents. Our findings demonstrate that both phosphonate-modified MSN-PEI (1.8 kDa) and MSN-PEI (10 kDa) efficiently bind and protect nucleic acids from enzymatic degradation, with the 1.8 kDa variant offering comparable delivery efficacy and enhanced biocompatibility. Our results validate MSN-PEI (1.8 kDa) as a promising carrier for safe and effective RNA delivery and highlight the therapeutic potential of codelivering RNA nanostructures with chemotherapeutics. This platform offers a versatile foundation for developing precision nanomedicines aimed at overcoming drug resistance in aggressive cancers such as triple-negative breast cancer.

## Materials and Methods

### Synthesis of AP-MSNs (Amino-Propyl Mesoporous Silica Nanoparticles)

AP-MSNs were synthesized using a modified protocol based on previously established methods. CTAB (0.78 g, 2.14 mmol) was dissolved in a mixture of ethanol (3.32 mL) and nanopure water (21.6 mL), followed by DEA (41.4 μL, 0.428 mmol). The solution was stirred at 60 °C for 30 min. APTES (7.64 μL, 32.6 μmol) was added, followed by dropwise addition of TEOS (2.19 mL, 9.80 mmol) over 5 min. The reaction mixture was stirred for 18 h at 60 °C. Nanoparticles were collected by centrifugation (13,000 rpm for 15 min), washed with ethanol (3×), and stored in ethanol.

Surfactant Template Extraction: MSNs were washed with a methanolic solution of 1 M HCl (10 mg MSNs in 1 mL of acid solution) and stirred at 60 °C for 10 h. After collecting and washing, a second acid wash was performed under identical conditions for 6 h. Surfactant-free AP-MSNs were washed with ethanol (3×) and stored in ethanol.

### Synthesis of MSN-cisPt (Cisplatin-Conjugated MSNs)

The MSN-cisPt were synthesized by conjugating the cisplatin prodrug (disuccinotocisplatin, compound 2) to amino-propylated mesoporous silica nanoparticles (AP-MSNs) via a coupling reaction mediated by 1-ethyl-3-(3-(dimethylamino)­propyl)­carbodiimide hydrochloride (EDC).

AP-MSNs (1000 mg) were dispersed in dimethyl sulfoxide (DMSO) (30 mL), and triethylamine (TEA) (210 μL, 151.8 mg, 1.5 mmol) was added. The dispersion was stirred for 30 min. Separately, compound 2 (400 mg, 0.75 mmol) and EDC (720 mg, 3.75 mmol) were dissolved in DMSO (20 mL). The two solutions were combined and stirred at room temperature for 24 h. The resulting cisPt-MSNs were collected via centrifugation, washed once with DMSO, twice with ethanol, and stored in ethanol. The nanoparticles were collected via centrifugation, washed, and stored in ethanol. The platinum content was determined by atomic absorption spectroscopy (AAS) using supernatants from the reaction and washing solutions.

### Synthesis of Phosphonate-Grafted MSNs (MSN-P/MSN-cisPt-P)

Phosphonate-functionalized MSNs were synthesized by postsynthetic grafting with trimethylphosphite (TPMP). AP-MSNs or cisPt-MSNs (200 mg) were dispersed in nanopure water (13 mL), and an aqueous solution of TPMP (113.5 μL, 0.2 mmol) was added (pH adjusted to 6–7). The mixture was stirred at 40 °C for 3 h. The nanoparticles were then collected via centrifugation and washed thrice with ethanol to obtain MSN-Por MSN-cisPt-P.

### PEI Coating of MSNs

Polyethylenimine (PEI, MW = 1.8 kDa, 10 kDa) coating was performed on MSN-P or MSN-cisPt-P to facilitate further functionalization. MSN-P or MSN-cisPt-P (100 mg) were dispersed in ethanol (40 mL), and a solution of PEI (0.03 M, 428.1 μL in ethanol) was added. The suspension was stirred for 1 h at room temperature. The nanoparticles were collected via centrifugation and washed thrice with ethanol to yield MSN-PEI (1.8 and 10 kDa) or MSN-cisPt-PEI (1.8 and 10 kDa).

To quantify the PEI coating, a ninhydrin assay was performed. MSN-PEI (1 mg) was dispersed in 4 mL of ethanol, and 1 mL of ninhydrin reagent (15 mg mL^–1^ in ethanol) was added. The reaction was stirred for 24 h, and the supernatant absorbance was measured at 575 nm using UV–vis spectroscopy. A calibration curve was generated using known concentrations of PEI.

### Synthesis of MSN-cisPt-PEI-Gem

MSN-cisPt-PEI-Gem were synthesized through a two-step process. Initially, MSN-PEI were conjugated to SPDP via NHS coupling, yielding MSN-SPDP. In the second step, the gemcitabine prodrug (compound 4) was conjugated to MSN-SPDP via disulfide exchange.

#### Step 1: Conjugation of SPDP to MSN-PEI

MSN-PEI or MSN-cisPt-PEI (30 mg) were dispersed in anhydrous acetonitrile (15 mL). SPDP (15 mg, 48 μmol) was added, and the reaction stirred at room temperature for 24–72 h. The nanoparticles were collected via centrifugation, washed thrice with ethanol, and stored in ethanol.

#### Step 2: Conjugation of Gem Prodrug to MSN-SPDP

The MSN-PEI-SPDP or MSN-cisPt-PEI-SPDP were reacted with compound 4 to form MSN-Gem. SPDP-21% cisPt-MSNs (30 mg) were dispersed in methanol (10 mL). A solution of compound 4 (30 mg, 85.4 μmol) in 5 mL methanol was added. The mixture was stirred for 72 h, and the resulting nanoparticles were collected via centrifugation, washed with methanol and ethanol, and stored. To achieve 18 wt % loading this step was repeated using an additional 20–25 mg of compound 4.

### Internalization and Lysosome Colocalization of Alexa Fluor 488-labeled DS DNA-MSNs-Confocal Microscopy

MDA-MB-231 cells (2.5 × 10^5^ cells per well) were plated in a 6-well plate containing cover glass and allowed to adhere for 24 h. Alexa Fluor 488-labeled DS DNA-MSN (10 nM of DS RNA) in DMEM was added to the cells and incubated for 24 h. The cells were rinsed twice with 1× DPBS and were incubated with 100 nM (prepared in 2 mL media) of LysoTracker Red dye for 2 h at 37 °C and then rinsed with 1× DPBS postincubation. The cells were further stained with DAPI for 20 min at room temperature. The cells were imaged using a 43× oil immersion objective (Leica Stellaris 8 Confocal).

### Binding Assays

To assess binding between MSNs and DNA, MSNs were mixed with a 27-base pair DNA or RNA duplex labeled with Alexa488 fluorophore. The mixing ratio was calculated based on the N/P ratio, where N represents the number of amine groups available on the MSN surface and P corresponds to the number of phosphate groups in the DNA duplex, ensuring proper electrostatic pairing. The mixture was prepared in a binding buffer containing 2 mM MgCl_2_ and 50 mM KCl and incubated at room temperature for 30 min to allow complex formation. Following incubation, the MSN–DNA complexes were immediately loaded onto a 1.5% agarose gel, and electrophoresis was performed at 200 V for 10 min to evaluate binding via mobility shift.

### Enzymatic Stability and Nuclease Protection Assay

MSN-PEI (1.8 kDa) were mixed with 27-bp DNA duplex labeled with Alexa488 fluorophore and Iowa Black Quencher (1 μM final) at N:P 10 as described above (see MSNs and DNA binding assays). DNase I (RNase-free DNase, Promega) was added to free quenched DNA and quenched DNA bound with MSNs and incubated for 30 min at 37 °C. Fluorescence measurements were obtained using a NanoDrop 3300 Fluorospectrometer (Thermo Scientific) and plotted via GraphPad Prism.

### GFP Silencing

MDA-MB-231/GFP cells were cultured at 37 °C with 5% CO_2_ in complete Dulbecco’s Modified Eagle Medium (DMEM) supplemented with 10% heat-inactivated fetal bovine serum (HI-FBS), 100 U/mL penicillin, and 100 μg/mL streptomycin. Approximately 50,000 cells were seeded into each well of a sterile flat-bottom 24-well plate 1 day prior to transfection. At 24 h postseeding, cells were treated with 50 μL of MSN complexed with GFP-targeting DS RNA. Following a 48-h incubation, fluorescence microscopy images were acquired using the Leica DMi8 imaging system. Cells were then harvested by adding 200 μL of 0.25% trypsin-EDTA per well, followed by neutralization with 300 μL of complete DMEM. The resulting cell suspensions were transferred into 1.5 mL microcentrifuge tubes and centrifuged at 300*g* for 5 min. Supernatants were discarded, and cell pellets were gently resuspended in 1× PBS containing 4% bovine serum albumin (BSA) and 0.2 mM EDTA.

Flow cytometry was performed using an Attune NxT Flow Cytometer equipped with a 488 nm blue laser. A population of 10,000 events was collected per treatment, and gating was based on the untreated cells’ condition. Quantification of fluorescence was performed using OVERTON analysis, which compares the fluorescence intensity distribution of treated samples to an untreated control. In this case, a leftward shift in GFP fluorescence indicated successful silencing of GFP expression in the cells following treatment with GFP-targeting DS RNA.

### Survivin Downregulation

To evaluate the efficiency of Survivin knockdown in MDA-MB-231 cells and compare the silencing performance between MSN-PEI (1.8 kDa) and MSN-PEI (10 kDa), MDA-MB-231 cells were seeded at a density of ∼150,000 cells per well in a 12-well flat-bottom Greiner plate and incubated for 24 h to allow proper adherence prior to transfection. Cells were then transfected with MSNs functionalized with either 1.8 kDa or 10 kDa PEI at N/P ratio 10. Each formulation was complexed with Survivin-targeting DS RNA and/or scrambled DS RNA at final concentrations of 25 and/or 50 nM.

### Nucleic Acid Nanoparticles (NANPs) Synthesis

All oligonucleotide sequences used in this study are provided in the Supporting Information. RNA sequences used for DS RNA assembly and DNA templates for RNA fiber transcription were obtained from Integrated DNA Technologies (IDT, Coralville, IA). These templates were PCR-amplified using MyTaq Mix (Bioline, London, U.K.), and the resulting products were purified using the DNA Clean and Concentrator kit (Zymo Research, Irvine, CA). In vitro transcription was carried out using T7 RNA polymerase in a reaction mixture containing 80 mM HEPES-KOH (pH 7.5), 2.5 mM spermidine, 50 mM DTT, 25 mM MgCl_2_, and 5 mM rNTPs. Reactions were incubated at 37 °C for 3.5 h, after which RQ1 RNase-free DNase (Promega, Madison, WI) was added to degrade DNA templates. Transcribed RNA was purified via 15% denaturing PAGE containing 8 M urea. RNA bands were visualized under UV light, excised, and eluted overnight in crush-and-soak buffer (300 mM NaCl, 89 mM Tris-borate, pH 8.2, and 2 mM EDTA). RNA was precipitated with 2× volumes of 100% ethanol at −20 °C for 3 h, followed by centrifugation at 14,000*g* for 30 mins. Pellets were washed twice with 90% ethanol (10 mins each), air-dried, and resuspended in endotoxin-free (ET-free) water.

All nucleic acid nanoparticles (NANPs) were assembled using a one-pot protocol in ET-free water. For RNA fiber NANPs, strands 1 and 2 were mixed at equimolar concentrations in ET-free water and heated to 95 °C for 2 mins. The samples were then snap-cooled on ice for 2 mins before adding 5× assembly buffer (89 mM Tris-borate, pH 8.2, 2 mM MgCl_2_, 50 mM KCl) to 20% of the final reaction volume. After buffer addition, samples were incubated at room temperature for 20 mins to allow complete assembly and then stored on ice until further use. Similarly, RNA cube NANPs were assembled using a one-pot method in ET-free water by mixing strands 1–6 at equimolar concentrations. Samples were heated to 95 °C for 2 mins, then cooled to 45 °C for 2 mins before the addition of 5× assembly buffer to 20% of the final volume. Following buffer addition, samples were incubated at 45 °C for 20 mins to ensure complete folding and then placed on ice until further use.

For DS RNA, strands 1 and 2 were also mixed at equimolar concentrations in ddiH_2_O, heated to 95 °C for 2 mins, and immediately followed by the addition of 5× assembly buffer to 20% of the final volume. Samples were incubated at room temperature for 20 mins to promote duplex formation and then transferred to ice for storage.

### Immunostimulation Assays

THP1-Dual and HEK-Lucia RIG-I cells were cultured according to InvivoGen’s recommended protocols under standard conditions (37 °C, 5% CO_2_). ∼100,000 THP1-Dual cells and ∼50,000 HEK-Lucia RIG-I cells were seeded per well in flat-bottom 96-well Greiner plates. On the following day, both cell lines were transfected with NANPs (final concentration of 10 nM per well) alongside their respective positive controls. For HEK-Lucia RIG-I cells, the positive control consisted of RNA cubes at 10 nM, which were complexed with Lipofectamine 2000 and incubated for 30 min at room temperature before transfection. For THP1-Dual cells, two separate positive controls were used depending on the assay: 2′,3′-cGAMP at 2 μg/mL for IRF activation (QUANTI-Luc assay), and PAM3CSK4 at 6 μg/mL for NF-κB activation (QUANTI-Blue assay). Both cGAMP and RNA cubes were incubated with L2K for 30 min at room temperature prior to transfection.

Following transfection, all cells were incubated for 24 h at 37 °C with 5% CO_2_. Post-treatment, immune pathway activation was assessed using QUANTI-Luc for IRF signaling and QUANTI-Blue for NF-κB signaling. All experiments were performed in biological triplicates, and fold induction values were normalized to untreated (cell-only) controls. Statistical analysis was ran through GraphPad Prism Software using a two-way ANOVA analysis.

### Cell Viability Assays

To evaluate the cytotoxic effects of the different MSN formulations, MDA-MB-231 cells were seeded on Day 1 and given 24 h to adhere. On Day 2, the cells were treated with different MSN formulations. After 48 h of treatment, the cells were washed with PBS and allowed to recover in fresh media for an additional 24 h. On Day 5, cell viability was assessed using an MTS assay after incubation for 150 min at 490 nm on a Tecan Spark microplate reader.

To conjugate the DS RNA/NANPs to MSN formulations for the cell viability assay, on Day 2, the DS RNA/NANP is incubated with the MSN formulation at a given concentration calculated from the N/P ratio (10) in assembly buffer for 45 min at 4 °C. Following incubation, the DS RNA/NANP coated MSN formulation was centrifuged for 5 min at 9000 rpm. The pellet is further dispersed in DMEM media followed by addition into the 96-well plates (100 μL each). All experiments were performed in biological triplicates, and fold induction values were normalized to untreated (cell-only) controls. Statistical analysis was run through Origin 2025 Software using a one-way ANOVA analysis.

### 3D Spheroid Formation and Treatment

3D spheroids of MDA-MB-231 cells were formed using a 96-well ultralow attachment format. Briefly, each well was coated with 60 μL of 1% (w/v) agarose gel to prevent cell adhesion. After the gel solidified, ∼10,000 cells were seeded per well in complete growth medium and centrifuged at 300 *g* for 5 min to promote spheroid formation. The spheroids were incubated under standard culture conditions (37 °C, 5% CO_2_) and allowed to grow for 4 days prior to treatment. On day 4, spheroids were treated with their respective formulations and imaged 24 h post-treatment using a Leica DMi8 fluorescence microscope. All spheroid experiments were performed following the same procedure.

## Supplementary Material



## Notes

Synthesis and characterization of cisplatin and gemcitabine prodrugs, physicochemical characterization of MSN materials, binding assay using agarose gel, drug-response plots for control groups, Western blot for control groups, cytotoxicity graphs for combined therapies (PDF)
