# Supplementary material for: Modular Design of Mesoporous Silica Nanoparticles Enables Bioimaging, Dual Chemotherapy, and Combinatorial Gene Silencing in Triple-Negative Breast Cancer
Source: ACS Appl Mater Interfaces. 2025 Dec 15;17(51):68925–38. doi: 10.1021/acsami.5c15253 (PMC12754758; doi:10.1021/acsami.5c15253)
Supplement: Supplementary file 1 [file am5c15253_si_001.pdf]

Supporting Information

Modular design of mesoporous silica nanoparticles enables bioimaging, dual chemotherapy, and combinatorial gene silencing in triple-negative breast cancer

Laura P. Rebolledo[#1,2], Punnya Anil Kumar Jeeja[#1,2], Leyla Danai[#1,2], Tamanna Binte Huq[1,2], Kirill A. Afonin[1,2,3*], and Juan L. Vivero-Escoto[1,2,3*]

1 – Department of Chemistry, University of North Carolina at Charlotte, Charlotte, NC 28223, USA.
2 – Chemistry and Nanoscale Science Program, University of North Carolina at Charlotte, Charlotte, NC 28223, USA.
3 – Center for Biomedical Engineering and Science, University of North Carolina at Charlotte, Charlotte, NC 28223, USA.

#-these authors contributed equally to this project
*- correspondence to Juan L. Vivero-Escoto at jviveroe@charlotte.edu and Kirill A. Afonin at kafonin@charlotte.edu.

Sequences Used in This Study

Survivin DS
5’ pGGACCACCGCAUCUCUACAUUCAAG
5’ CUUGAAUGUAGAGAUGCGGUGGUCCUU

BCL-2 DS:
5’ pGUACAUCCAUUAUAAGCUGUCGCAG
5’ CUGCGACAGCUUAUAAUGGAUGUACUU

GFP DS:
5’ pCAUUAACGAGCUGCUUAAUGACGA
5’ CGGUGGUGCAGAUGAACUUCAGGGUCA

Al488-DNA Duplex:
5’ CGGTGGTGCAGATGAACTTCAGGGTCA
5’ TGACCCTGAAGTTCATCTGCACCACCG/3AlexF488N/

Al488-DNA Duplex with Iowa Black Quencher:
5’ CGGTGGTGCAGATGAACTTCAGGGTCA/3IAbRQSp/
5’ TGACCCTGAAGTTCATCTGCACCACCG/3AlexF488N/

Survivin Fiber:
A: 5’ GGGAAUCCAAGGAGGCAGGAUUCCCGUCACAGAAGGAGGCACUGUGAC
B_Survivin: 5’
GGGAACGUAAGCCUCCAACGUUCCCGGAUGCUAAGCCUCCAAGCAUCCUUUCUUGAAUGUAGAG
AUGCGGUGGUCCUU
Survivin sense: 5’ pGGACCACCGCAUCUCUACAUUCAAG

BCL-2 Fiber:
A_BCL2: 5’
GGGAAUCCAAGGAGGCAGGAUUCCCGUCACAGAAGGAGGCACUGUGACUUUCUGCGACAGCUUA
UAAUGGAUGUACUU
B: 5’ GGGAACGUAAGCCUCCAACGUUCCCGGAUGCUAAGCCUCCAAGCAUCC
BCL-2_sense: 5’ pGUACAUCCAUUAUAAGCUGUCGCAG

Survivin/BCL-2 Fiber:
A_BCL2: 5’
GGGAAUCCAAGGAGGCAGGAUUCCCGUCACAGAAGGAGGCACUGUGACUUUCUGCGACAGCUUA
UAAUGGAUGUACUU
B_Survivin: 5’
GGGAACGUAAGCCUCCAACGUUCCCGGAUGCUAAGCCUCCAAGCAUCCUUUCUUGAAUGUAGAG
AUGCGGUGGUCCUU
BCL2_sense: 5’ pGUACAUCCAUUAUAAGCUGUCGCAG
Survivin_sense: 5’ pGGACCACCGCAUCUCUACAUUCAAG

RNA cube:
1 - GGCAACUUUGAUCCCUCGGUUUAGCGCCGGCCUUUUCUCCCACACUUUCACG
2 - GGGAAAUUUCGUGGUAGGUUUUGUUGCCCGUGUUUCUACGAUUACUUUGGUC

3 - GGACAUUUUCGAGACAGCAUUUUUUCCCGACCUUUGCGGAUUGUAUUUUAGG
4 - GGCGCUUUUGACCUUCUGCUUUAUGUCCCCUAUUUCUUAAUGACUUUUGGCC
5 - GGGAGAUUUAGUCAUUAAGUUUUACAAUCCGCUUUGUAAUCGUAGUUUGUGU
6 - GGGAUCUUUACCUACCACGUUUUGCUGUCUCGUUUGCAGAAGGUCUUUCCGA

Extended Materials and Methods

Dynamic Light Scattering (DLS) and Zeta Potential Analysis. Hydrodynamic size measurements of mesoporous silica nanoparticles (MSNs) were carried out using a Zetasizer Nano instrument (Malvern Instruments). MSNs were dispersed at a concentration of 0.1 mg mL^{-1} in deionized water, phosphate-buffered saline (PBS, 1 mM, pH 7.4), or complete cell culture medium supplemented with 10% fetal bovine serum (FBS). The dispersions were sonicated for 10 minutes prior to analysis. Zeta (ζ) potential measurements were conducted using the same instrument and MSN concentration in PBS (1 mM, pH 7.4).

Surface Area and Pore Size Analysis. MSNs (20-25 mg) were dried under vacuum and degassed overnight at 80 °C to remove physisorbed solvents. Nitrogen adsorption-desorption isotherms were obtained using a NOVA 2200e surface area and pore size analyzer (Quantachrome). Specific surface area was calculated using the Brunauer-Emmett-Teller (BET) method, while pore volume and pore diameter were determined using the Barrett-Joyner-Halenda (BJH) analysis.

MSN-Gem (18%). To synthesize 18% MSN-Gem MSN-PEI-SPDP (30 mg) were dispersed in 10 mL of methanol. A solution of compound (4) (30 mg, 85.4 µmol) in 5 mL of methanol was added to the dispersion, and the reaction mixture was stirred at room temperature for 72 h. To achieve 22% loading, the reaction was repeated twice, sequentially adding 20–25 mg and 15 mg of compound (4) (Gemcitabine step 2 product) to the reaction mixture. After the final conjugation step, the nanoparticles were collected via centrifugation, washed once with methanol, followed by a wash with ethanol, and stored for further use.

Quantification of Gem Conjugation. The conjugation efficiency of Gem was determined by measuring the amount of 2-thiopyridine, a byproduct of the disulfide exchange reaction between compound 4 and MSN-PEI-SPDP. The supernatants from the reaction and washing steps were analyzed for 2-thiopyridine content using UV-Vis spectroscopy at 357 nm. This systematic synthesis provides tunable ratios of gemcitabine and cisplatin loading on MSNs, optimizing their potential for combined cancer therapy.

Survivin Downregulation. At 72 h post-transfection, protein was extracted for western blot analysis. To begin, the culture media was carefully aspirated from each well, and the cells were washed once with 100 µL of 1X PBS. Following aspiration of the PBS, 150 µL of 0.25% trypsin-EDTA was added to each well and incubated at room temperature for 3–5 minutes to facilitate detachment of adherent cells. Trypsinization was neutralized by adding 150 µL of complete DMEM. The resulting 300 µL cell suspension was transferred to a 1.5 mL tube and centrifuged at 800 × g for 5 min at 4 ºC. The supernatant was gently aspirated, and the cell pellet was washed with 300 µL of ice-cold 1X PBS, followed by another centrifugation step at 800 × g for 5 min at 4 °C. The PBS was aspirated with caution to avoid disturbing the cell pellet. To lyse the cells, 100 µL of ice-cold TX-100 lysis buffer was added directly to the pellet, and samples were incubated

on ice at 4 ºC for 30 min. After lysis, samples were sonicated for 10–15 secs and returned to ice for an additional 5 min. Cell debris was pelleted by centrifugation at 12,000 x g for 20 min at 4 °C. The resulting supernatant containing total cellular protein was carefully transferred to a fresh tube and kept on ice until protein quantification using the Pierce BCA Protein Assay Kit. Per the manufacturer's instruction, the working reagent was prepared by mixing 3 mL of Reagent A with 60 µL of Reagent B (1:50 ratio). On a 96-well plate, 200 µL of freshly prepared BCA reagent was added to each well, followed by 25 µL of each protein sample in duplicate. The plate was incubated at 37 °C for 45 min, after which absorbance (at 562 nm) was measured using a plate reader to determine protein concentration.

Once the protein concentration of each treatment group was determined via BCA assay, western blot analysis was performed to assess protein expression. Protein samples were prepared for gel electrophoresis by mixing equal volumes of cell lysate with 4X Laemmli sample buffer and boiling the mixtures at 95 °C for 5 min to denature the proteins. Samples were then briefly centrifuged to collect condensation and loaded onto a 10% SDS-PAGE gel. Electrophoresis was carried out at a constant voltage of 100 V for approximately 1 h in running buffer (25 mM Tris, 192 mM glycine, 0.1% SDS). During electrophoresis, 6 pieces of Whatman filter paper and one nitrocellulose membrane were soaked in cold transfer buffer (25 mM Tris, 192 mM glycine, 20% methanol) in preparation for protein transfer.

Following gel electrophoresis, proteins were transferred onto the nitrocellulose membrane using a semi-dry transfer system. The transfer stack was assembled in the following order: three layers of pre-wetted Whatman paper, the nitrocellulose membrane, the SDS-PAGE gel, and three additional layers of Whatman paper. Protein transfer was conducted at a constant current of 200 mA for 45 min. After transfer, the membrane was sectioned as needed and blocked with 5% non-fat dry milk in 1X TBST (Tris-buffered saline with 0.1% Tween-20) for 1 h at room temperature. The membrane was then washed three times with 1X TBST for 10 min each to remove excess blocking solution. Primary antibody diluted in 1X TBST with 5% BSA or milk was added and incubated overnight at 4 °C with gentle rocking. The following day, the membrane was washed three times in 1X TBST, 10 min increments, before being incubated with an HRP-conjugated secondary antibody for 1 h at room temperature. A final set of three 10 min washes with 1X TBST was performed prior to chemiluminescent detection. Chemiluminescent western blotting was used to visualize protein bands. To prepare the chemiluminescent substrate, 12 mL of double-distilled water was mixed with 1 mL of reagent A and 1 mL of reagent B. The membrane was incubated in the substrate solution for 5 minutes before imaging using the ChemiDoc imaging system.

Synthesis of Prodrugs

Synthesis of Cisplatin Prodrug. The synthesis of cisplatin (IV) prodrug, disuccinotocisplatin, involves a two-step process adapted from established methodologies with slight modifications to optimize the procedure.

Step 1: Oxidation of Cisplatin to Dihydroxycisplatin(1)

The initial step involves oxidizing cisplatin to dihydroxycisplatin. Cisplatin (200 mg, 0.67 mmol) was dissolved in nanopure water (9 mL, pH 7), and hydrogen peroxide (30 wt%, 1 mL) was added. The reaction mixture was stirred at 70 °C for 5 h under a nitrogen atmosphere. The mixture was then cooled to room temperature and stirred overnight. The product was washed with ice-cold water and ethanol, yielding dihydroxycisplatin.

Step 2: Reaction with Succinic Anhydride to Yield Disuccinotocisplatin (2)

Dihydroxycisplatin (100 mg, 0.3 mmol) was dissolved in 4 mL of dimethyl sulfoxide (DMSO), followed by the addition of succinic anhydride (120.4 mg, 1.2 mmol). The reaction mixture was stirred at 70 °C for 24 h in the dark. The product was vacuum-dried, washed with cold acetone, and collected as disuccinotocisplatin. The reaction likely proceeds through the formation of an amide bond between dihydroxycisplatin and succinic anhydride.

Characterization:

- NMR Spectroscopy
  - ^{1}H NMR (300 MHz, DMSO-d6): δ 2.61-2.77 (m, 2H), 2.21-2.32 (m, 2H).
  - ^{13}C NMR (300 MHz, DMSO-d6): δ 180.1, 174.3, 30.9, 29.1.
- IR Spectroscopy
  - Step 1 Yield: 45 wt%. FT-IR (cm^{-1}): 3517 (O-H), 3267 (N-H).
  - Step 2 Yield: 65 wt%. FT-IR (cm^{-1}): 3457 (O-H), 3262 (N-H), 2920 (C-H), 1705 (C=O).

These analyses confirm the structure and successful synthesis of disuccinotocisplatin.

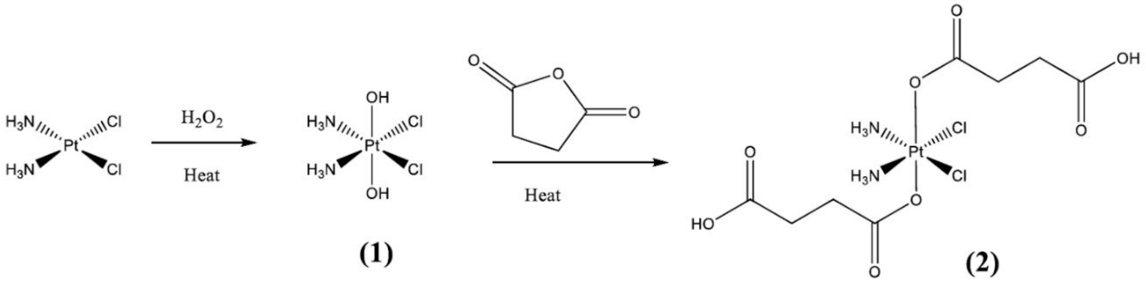

Scheme S1: Outlined reaction steps and associated conditions employed for the chemical synthesis of the cisplatin prodrug.

Synthesis of Gemcitabine Prodrug. The gemcitabine (Gem) prodrug synthesis was carried out in two steps based on literature protocols with modifications.

Step 1: Synthesis of 3-Tritylthio-Gemcitabine (3)

S-trityl-mercaptopropionic acid (136 mg, 0.4 mmol) and TBTU (143 mg, 0.44 mmol) were dissolved in anhydrous DMF (2 mL), followed by the addition of gemcitabine hydrochloride (200 mg, 0.64 mmol). DIPEA (244 µL, 1.58 mmol) was added, and the reaction stirred at room temperature for 72 hours. The product was precipitated with cold brine, filtered, and dried under reduced pressure. After drying, one time wash with nanopure water confirms the removal of excess salt from the step 1 product.

Yield: 76 wt%.

- ^{1}H NMR (300 MHz, MeOD-d4): δ 7.78 (d, 1H, J = 3.6 Hz), 7.21-7.23 (m, 16H), 6.25-6.28 (t, 1H, J = 8.6 Hz), 4.89-5.55 (d, 1H, J = 10.8 Hz), 4.51-4.67 (m, 1H), 4.88-4.95 (m, 2H), 4.20-4.25 (m, 1H), 2.11-2.23 (m, 2H), 2.45-2.89 (m, 2H).

- ^{13}C NMR (300 MHz, MeOD-d4): δ 177.43, 156.76, 176.31, 142.65, 134.28, 127.67, 126.45, 92.31, 87.94, 83.15, 70.42, 67.89, 59.21, 37.76, 27.03.

• FT-IR (cm^{-1}): 3320 (O–H), 2950 (C–H), 1735 (C=O), 1640 (C=C), 1570 (C=N), 1140 (C–O)

Step 2: Synthesis of Compound (4)

3-Tritylthio-gemcitabine (80 mg, 0.14 mmol) was dissolved in a 1:1 mixture of TFA and DCM (2 mL). Triethylsilane (0.5 mL, 3.0 mmol) was added, and the mixture stirred at room temperature for 1 hour. The product was dried with Rotavap, sonicated and vortexed with diethyl ether (5×5 mL), and dried again to yield compound (4).

Yield: 18 wt%.

- ^1H NMR (300 MHz, MeOD-d4): δ 8.32 (d, 1H, J = 6.6 Hz), 7.48–7.42 (t, 1H, J = 7.5 Hz), 6.26–6.20 (t, 1H, J = 7.2 Hz), 4.28–4.22 (m, 1H), 3.95–3.91 (m, 2H), 2.77–2.72 (m, 4H).
- ^{13}C NMR (300 MHz, MeOD-d4): δ 172.48, 144.69, 128.97, 96.85, 85.33, 61.92, 58.44, 37.72.
- FT-IR (cm^{-1}): 3265 (O–H), 2940 (C–H), 1725 (C=O), 1645 (C=C), 1560 (C=N), 1142 (C–O)

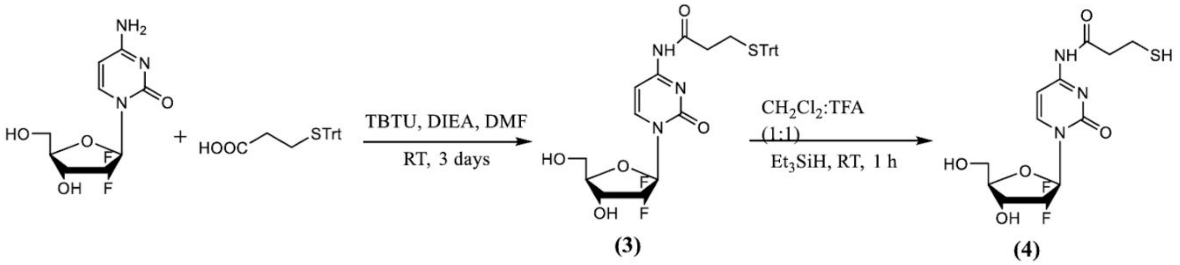

Scheme S2: Reaction pathway and conditions used in the synthesis of the Gem prodrug.

Supporting Tables

MSN Formulation	Percentage of cisPt (%wt)	Percentage of Gem (%wt)	EC_{50} in (µg/mL) of MSN	EC_{50} in terms of cisPt (µM)	EC_{50} in terms of Gem (µM)
MSN-PEI	-	-	>1000	-	-
MSN-cisPt-PEI	21.1 ± 1.2 %wt (n=4)	-	538.7 ± 9.2	304.2	-
MSN-PEI-Gem	-	18.7 ± 0.7 % wt (n=8)	38.5 ± 3.3	-	23.1
MSN-cisPt-PEI-Gem	21.1 ± 1.2 %wt (n=4)	18.7 ± 0.7 % wt (n=8)	21.1 ± 5.3	11.9	12.6

Table S1: EC_{50} values of MSN formulations from MTS cytotoxicity experiments.

Concentration of BCL2/Survivin DS RNAs (nM)	Concentration of MSN-PEI (µg/mL)	Concentration of MSN-cisPt-PEI-Gem (µg/mL)	Concentration of cisPt in MSN-cisPt-PEI-Gem ((µM)	Concentration of Gem in MSN-cisPt-PEI-Gem ((µM)
1	0.505	0.66	0.46	0.28
10	5.05	6.6	4.62	2.77
15	7.6	9.9	6.93	4.16
25	12.6	16.5	11.55	6.94
50	25.2	32.9	23.03	13.83
100	50.5	65.8	46.05	27.66

Table S2: Amounts of MSNs (MSN-PEI, MSN-cisPt-PEI-Gem) and the corresponding drug (cisPt, Gem) concentrations added to BCL2/Survivin DS RNAs at N/P-10 for cytotoxicity experiments.

Concentration of BCL2 DS RNAs (nM)	Cell viability of MSN-PEI (µg/mL)	Cell viability of MSN-cisPt-PEI-Gem (µg/mL)	Cell Viability of MSN-PEI-DS RNA ((µM)	Cell viability of MSN-cisPt-PEI-Gem-DS RNA (µg/mL)
10	95.4 ±12.4	74.5 ± 8.9	65.3 ± 9.8	49.2 ± 5.3
15	102.4 ± 9.5	74.2 ± 5.3	68.2 ± 9.3	48.6 ± 4.3
25	47.6 ± 4.4	60.5 ± 9.9	67.4 ± 8.6	47.6 ± 4.4
50	110.1 ± 9.8	65.9 ± 8.9	68.0 ± 9.1	44.5 ± 2.9
100	113.4 ± 8.2	26.5 ± 9.9	67.8 ± 7.6	32.0 ± 8.0

Table S3: Cell viability data of BCL2 DS RNA with MSN-PEI and MSN-cisPt-PEI from MTS cytotoxicity experiments.

Concentration of Survivin DS RNAs (nM)	Cell viability of MSN-PEI (μg/mL)	Cell viability of MSN-cisPt-PEI-Gem (μg/mL)	Cell Viability of MSN-PEI-DS RNA ((μM)	Cell viability of MSN-cisPt-PEI-Gem-DS RNA (μg/mL)
10	104.9 ± 13.4	73.3 ± 10.1	63.3 ± 4.6	52.4 ± 5.2
15	105.6 ± 11.8	66.2 ± 8.8	66.2 ± 5.1	44.8 ± 9.5
25	95.6 ± 4.2	57.8 ± 4.3	62.5 ± 6.6	50.6 ± 6.2
50	95.2 ± 6.4	40.5 ± 5.4	64.3 ± 7.9	44.4 ± 6.6
100	97.3 ± 10.7	24.65 ± 1.9	67.65 ± 4.1	39.4 ± 4.0

Table S4: Cell viability data of Survivin DS RNA with MSN-PEI and MSN-cisPt-PEI from MTS cytotoxicity experiments.

Concentration of BCL2/Survivin fNAs (nM)	Concentration of MSN-PEI (μg/mL)	Concentration of MSN-cisPt-PEI-Gem (μg/mL)	Concentration of cisPt in MSN-cisPt-PEI-Gem ((μM)	Concentration of Gem in MSN-cisPt-PEI-Gem ((μM)
1	1.45	1.89	1.32	0.79
10	14.5	18.9	13.23	7.95

Table S5: Amounts of MSNs (MSN-PEI, MSN-cisPt-PEI-Gem) and the corresponding drug (cisPt, Gem) concentrations added to BCL2/Survivin fNAs for cytotoxicity experiments.

Concentration of BCL2 fNA (nM)	Cell viability of MSN-PEI (μg/mL)	Cell viability of MSN-cisPt-PEI-Gem (μg/mL)	Cell Viability of MSN-PEI-DS RNA ((μM)	Cell viability of MSN-cisPt-PEI-Gem-DS RNA (μg/mL)
1	103.6 ± 13.4	83.2 ± 8.9	72.5 ± 13.2	64.9 ± 7.3
10	94.1 ± 7.1	60.1 ± 10.9	63.4 ±1 2.8	53.3 ± 6.5

Table S6: Cell viability data of BCL2 fNA with MSN-PEI and MSN-cisPt-PEI from MTS cytotoxicity experiments.

Concentration of Survivin fNA (nM)	Cell viability of MSN-PEI (μg/mL)	Cell viability of MSN-cisPt-PEI-Gem (μg/mL)	Cell Viability of MSN-PEI-DS RNA ((μM)	Cell viability of MSN-cisPt-PEI-Gem-DS RNA (μg/mL)
1	103.1 ± 10.0	83.4 ± 7.9	77.0 ± 9.7	70.3 ± 8.1
10	91.9 ± 5.9	68.5 ± 7.3	69.9 ± 7.3	59.9 ± 4.4

Table S7: Cell viability data of Survivin fNA with MSN-PEI and MSN-cisPt-PEI from MTS cytotoxicity experiments.

Supporting Figures

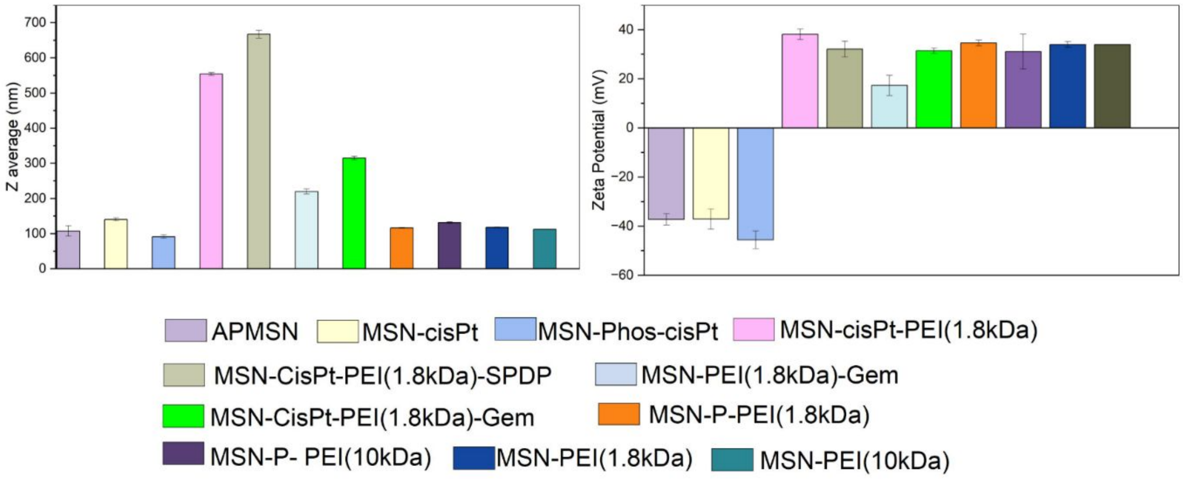

Fig S1: (A) Zeta potential and (B) Z-average of the different versions of MSN nanoparticles using Dynamic Light Scattering (DLS).

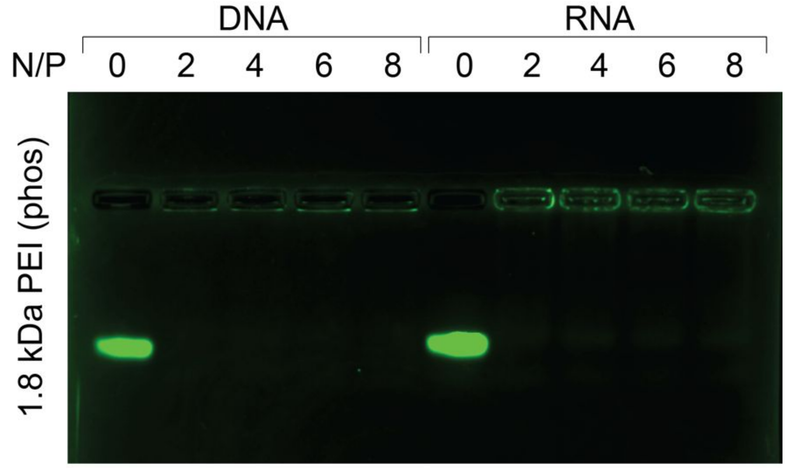

Fig S2: Agarose gel comparing the binding of Alexa488-labeled 27-bp DNA and RNA duplexes with phosphonate-modified (phos) MSNs functionalized with 1.8 kDa or 10 kDa PEI at varying N/P ratios.

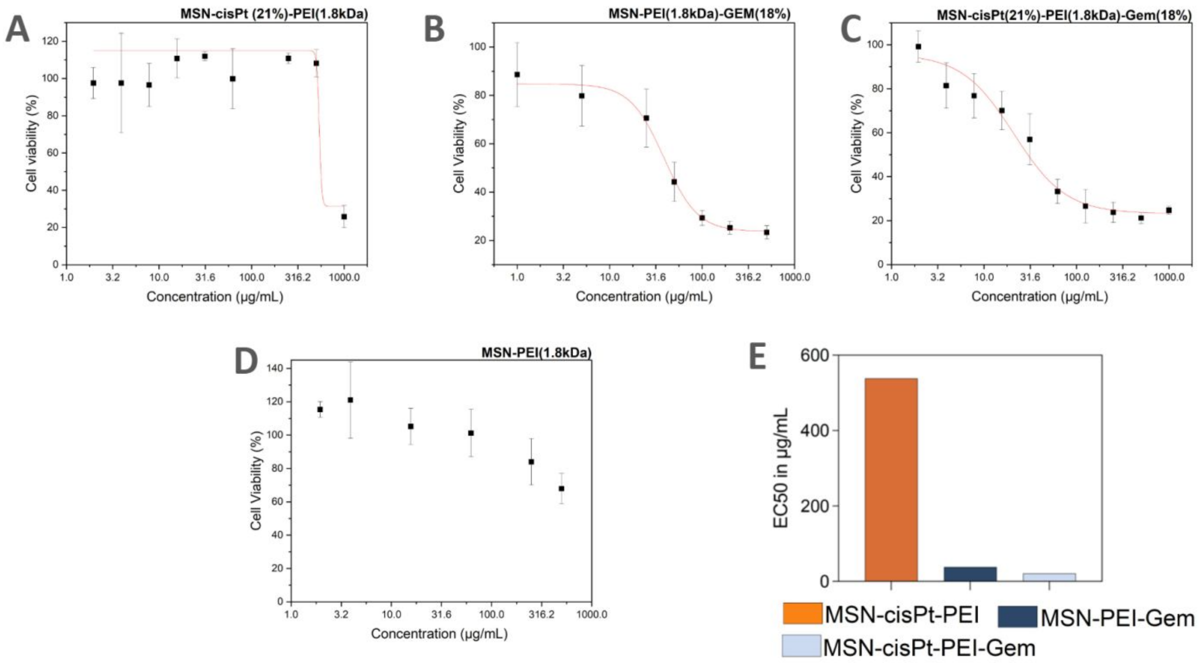

Fig S3: Drug-response plots for (A) MSN-cisPt-PEI, (B) MSN-PEI-Gem, (C) MSN-cisPt-PEI-Gem, and (D) MSN-PEI and EC50 value of MSN-cisPt-PEI, MSN-PEI-Gem and MSN-cisPt-PEI-Gem from the drug response plots

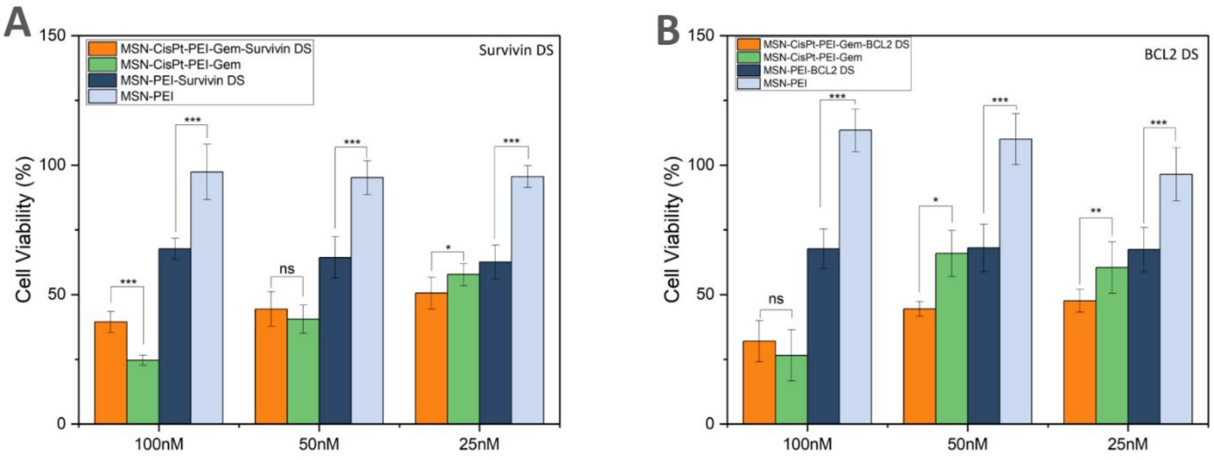

Fig S4: Cytotoxicity of (**A**) BCL2 DS RNA-loaded MSN-cisPt-PEI-Gem and MSN-PEI; and (**B**) Survivin DS RNA-loaded MSN-cisPt-PEI-Gem and MSN-PEI. Error bars represent mean ± SD from three biological replicates (n=3).

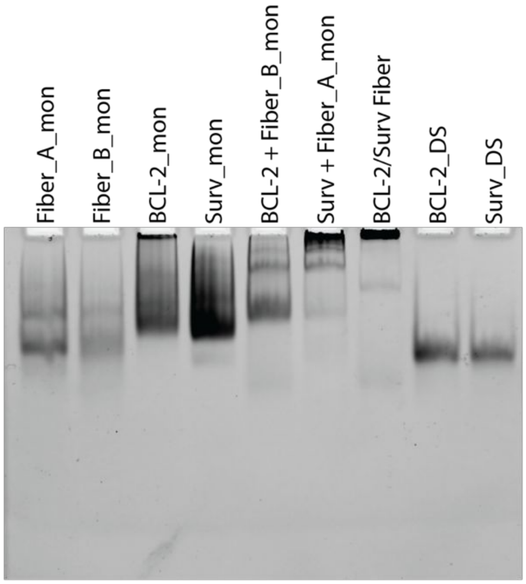

Fig S5: Ethidium bromide-stained native PAGE confirming the formation of DS RNAs and fiber NANPs assemblies used in this study.

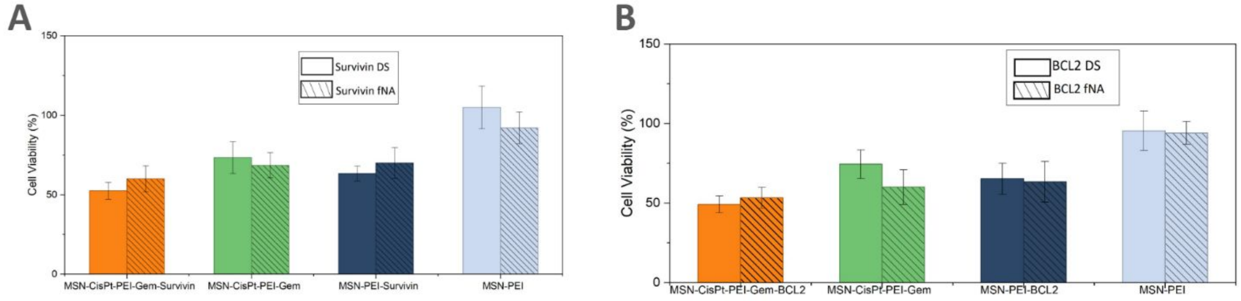

Fig S6: Cytotoxicity of DS RNA (unstriped) compared with fNAs (striped) -loaded MSN at 10 nM (**A**) Survivin (**B**) BCL2. Error bars represent mean ± SD from three biological replicates (n=3).

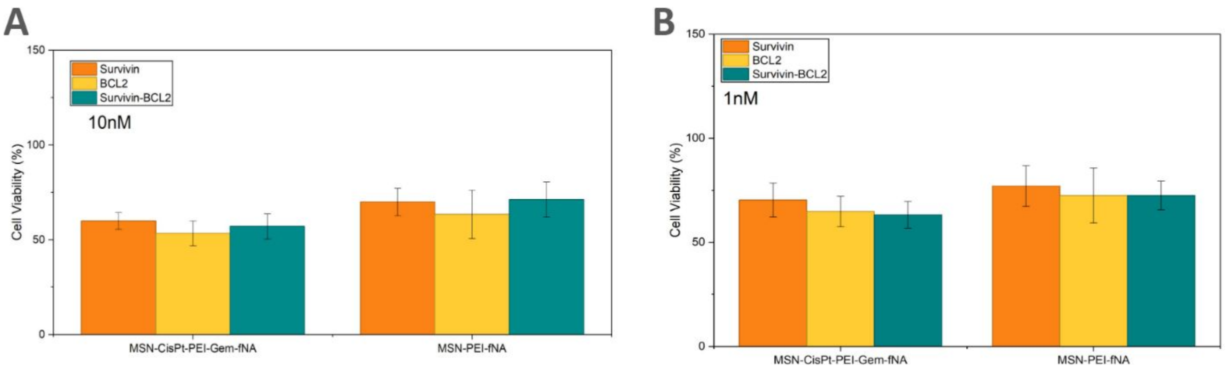

Fig S7: Cytotoxicity of Surv-BCL2-fNA compared with Survivin and BCL2 fNAs at (**A**) 10 and (**B**) 1 nM. Error bars represent mean ± SD from three biological replicates (n=3).

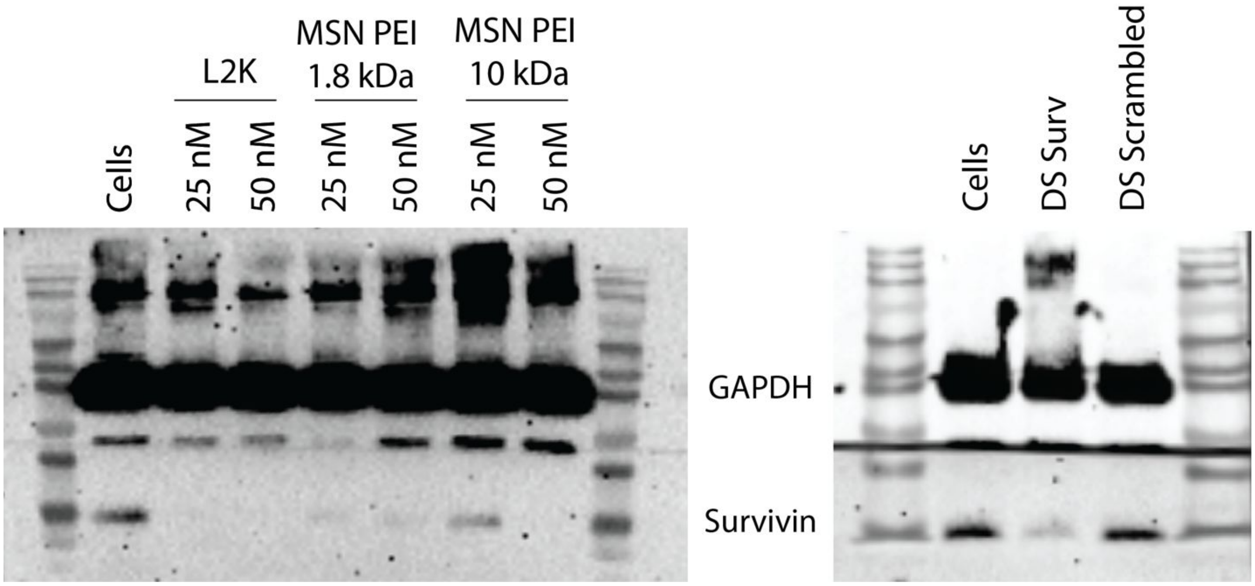

Fig S8: Full Western blot images corresponding to Figure 4C and Figure 4D. Blots show Survivin protein expression in MDA-MB-231 cells following the indicated treatments. GAPDH was used as a loading control. Molecular weight markers are included for reference.

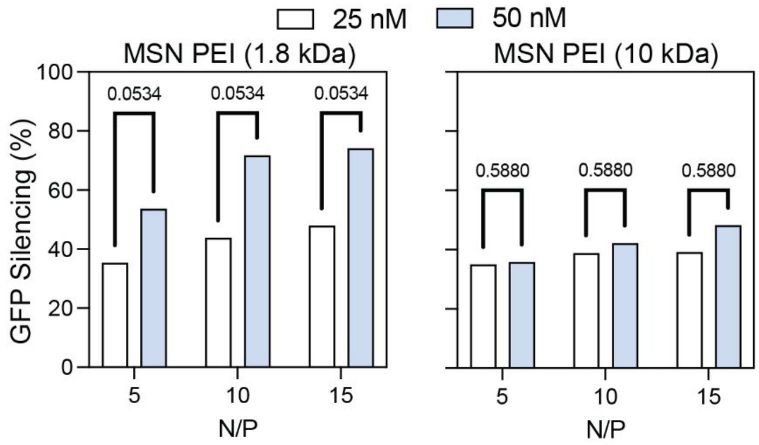

Fig S9: GFP silencing was assessed in MDA-MB-231 cells that overexpress GFP, with percentage knockdown shown for MSN-PEI 1.8 kDa and 10 kDa at varying N/P ratios and siRNA concentrations of 25 and 50 nM (n=1).
